# Implementing the donor–acceptor approach in electronically conducting copolymers *via* electropolymerization

**DOI:** 10.1039/d2ra01176j

**Published:** 2022-04-20

**Authors:** R. M. Gamini Rajapakse, Davita L. Watkins, Tharindu A. Ranathunge, A. U. Malikaramage, H. M. N. P. Gunarathna, Lahiru Sandakelum, Shane Wylie, P. G. P. R. Abewardana, M. G. S. A. M. E. W. D. D. K. Egodawele, W. H. M. R. N. K. Herath, Sanjaya V. Bandara, Daniel R. Strongin, Nuwan Harsha Attanayake, Dhayalan Velauthapillai, Benjamin R. Horrocks

**Affiliations:** Department of Chemistry, University of Peradeniya Peradeniya 20400 Sri Lanka rmgr@pdn.ac.lk; Department of Chemistry, The University of Mississippi 322 Coulter Hall University MS USA; Department of Chemistry, College of Science and Technology, Temple University 1901 N. 13th Street Philadelphia PA 19122-6081 USA; US National Renewable Energy Laboratory (NREL) 1513 Denver West Parkway Golden CO 80401 USA; US National Renewable Energy Laboratory (NREL) 1513 Denver West Parkway Golden CO 80401 USA; Department of Computer Science, Electrical Engineering and Mathematical Sciences, Western Norway University of Applied Sciences Bergen Kronstad D412 Norway; School of Natural and Environmental Sciences, Newcastle University Newcastle Upon Tyne NE1 7RU UK

## Abstract

Electropolymerization has become a convenient method for synthesizing and characterizing complex organic copolymers having intrinsic electronic conductivity, including the donor (D)–acceptor (A) class of electronically conducting polymers (ECPs). This review begins with an introduction to the electrosynthesis of common second-generation ECPs. The information obtainable from electroanalytical studies, charge carriers such as polarons (positive and negative) and bipolarons (positive and negative) and doping will be discussed. The evolutionary chain of ECPs is then presented. ECPs comprising electron-rich D and electron-deficient A moieties have been shown to possess intrinsic electronic conductivity and unique optical and electronic properties. They are third generation ECPs and electropolymerization of mixtures of D and A leads to stoichiometrically controlled block copolymers. These D–A type ECPs are discussed on the basis of selected representative materials. Since the discovery of electropolymerization as a powerful tool to synthesize copolymers of conjugated monomers with a pre-determined ratio of D and A repeat units present in the polymer, the field of D–A type ECPs has grown considerably and the literature available since 2004 to 2021 is summarized and tabulated. Electronic and optical properties of the materials determined by computational chemistry are presented. The data obtained from electrochemical and optical methods are compared with those obtained from computational methods and reasons for discrepancies are given. The literature on the concept of electropolymerization extended to synthesizing triblock and many-block copolymers is reviewed. Finally, applications of D–A polymers in optoelectronic devices (organic solar cells and field-effect transistors) and in bio-imaging are explained quoting appropriate examples.

## Abstract 2

Electropolymerization has become a convenient method for synthesizing and characterizing complex organic copolymers having intrinsic electronic conductivity, including the donor (D)–acceptor (A) class of electronically conducting polymers (ECPs).

## Graphical abstract

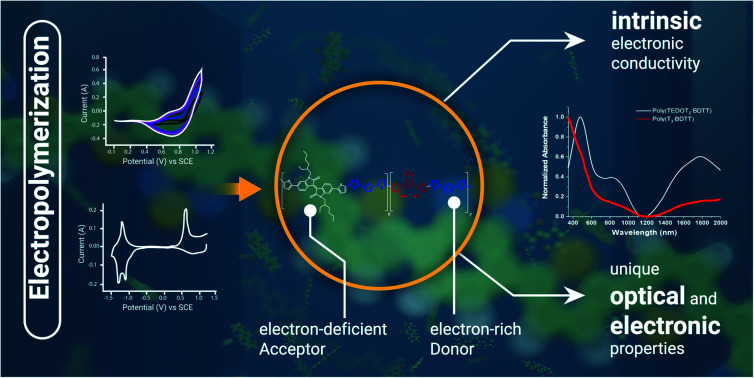

## 1. Introduction

### 1.1 Basics of electropolymerization leading to conducting polymers

#### 1.1.1 Basics of electropolymerization leading to 2^nd^ generation conducting polymers

**Fig. 1:**
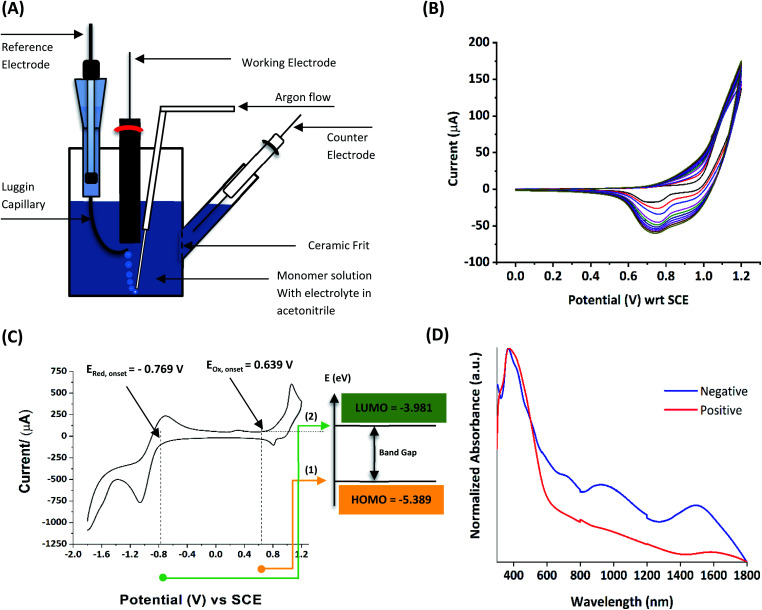
(A) The electropolymerization setup showing all necessary components (B) the typical cyclic voltammograms (CVs) obtained in the polymerization of terthiophene in acetonitrile solution containing 0.10 M tetrabutylammonium hexafluorophosphate/CH_3_CN background electrolyte (BGE) in the potential range from 0 V to 1.0 V wrt the saturated calomel reference electrode, at the scan rate of 100 mV s^−1^ (C) the corresponding energy level diagram showing energies of HOMO and LUMO levels of polyterthiophene with respect to the vacuum level. (D) The UV-visible-NIR spectrum of polyterthiophene. Authors own work. This figure has been reproduced from ref. 42 with permission from RSC *Journal of Material Chemistry C*, copyright 2019 and ref. 71 with permission from Chemistry Europe, copyright 2020.

Electropolymerization is a convenient and simple method used to synthesize common 2nd generation electronically conducting polymers (EPCs) such as polypyrrole (PP),^1,2^ polyaniline (PAN),^3–5^ polythiophene (PT),^6,7^ polyethylenedioxythiophene (PEDOT),^8^ and their derivatives. This type of polymerization offers an environmentally friendly approach since hazardous oxidants used in chemical polymerization are not required, and instead, electrons are added/removed by applying the required negative/positive potentials. Electropolymerization has a high atom efficiency also. It is generally carried out in a two-compartment cell, connected using a ceramic frit, both compartments filled with the background electrolyte (BGE) containing the monomer. The working electrode (WE, electrode at which the desired half-reaction occurs) and the reference electrode (RE) are placed in the main compartment. The RE is placed very close to the WE to minimize the *iR* potential drop arising due to current *i* passing through the interelectrode gap of resistance *R*, within the electrolyte solution. The counter electrode (CE) is often placed in a second compartment containing the same solution. The background electrolyte that is present in high concentration, compared to the concentration of the monomer, provides electrochemically inert ions to the solution to increase the ionic conductivity. Further, the high concentration of counterions in the BGE screens the charge on the electrode and changes in applied potential correspond to changes in the driving force for the electrooxidation reaction, which occurs at the working electrode/solution interface. The three electrodes are connected to the electrochemical analyser and the solution is degassed using a flow of an inert gas such as nitrogen or argon to remove dissolved oxygen that is electroactive within the potential ranges used in these experiments and may react with radical intermediates of the polymerization mechanism. During the potential application, a slow flow of the inert gas is maintained above the solution to prevent re-entry of air. The gas flow is not directed into the solution to avoid the convection introduced by the purging the gas. Then, the desired potential program is chosen, which can be either a constant potential capable of oxidatively/reductively polymerizing the monomer in the potentiostatic mode, or potential cycling within the chosen limits in the cyclic voltammetric (CV) mode. In the latter, the potential range is chosen such that at the lower potential, all the materials are electro-inactive and when the potential is swept from the lower limit to the upper limit, only the monomer and oligomers are electroactive. Alternatively, the polymerization can also be performed in galvanostatic mode, where a constant current is used to polymerize the monomer on the WE surface. The solvent used can be water if the monomer is soluble in it. In that case, a background electrolyte such as potassium perchlorate can be used since both the K^+^(aq) and ClO_4_^−^ (aq) ions are inert within the potential range chosen. Note, however, that water undergoes oxidation at a Pt electrode surface (standard electrode potential of H_2_O/O_2_ in the acidic medium at pH 0 is +1.23 V with respect to (wrt) the standard hydrogen gas electrode (SHE)).^9^ Therefore, the accessible positive potential is limited by the water oxidation and by H^+^(aq) reduction at negative potentials. In addition, water is a weak nucleophile that can attack the cation radicals of the monomer formed during oxidative polymerization, leading to undesired side reactions. In many cases, the monomers used are not readily soluble in water and such cases a polar non-aqueous solvent, such as acetonitrile, is commonly used. In this case, quaternary ammonium salts such as tetrabutylammonium tetrafluoroborate (TBATFB)^10^ or tetrabutylammonium hexafluorophosphate (TBAHFP)^10,11^ are used as the BGE since salts containing large cations and large anions are more soluble in polar non-aqueous solvents. Additionally, acetonitrile and the ions derived from quaternary ammonium salts are electrochemically inert^12^ in a much wider potential range. Hence, the electrochemical window (the potential range at which all the substances are electro-inactive) accessible is wider for acetonitrile than that for water. The use of inert solvents also avoids the possible side reactions. Fig. 1(A) shows the electropolymerization setup and Fig. 1(B) shows the typical CV obtained in the polymerization of terthiophene in acetonitrile solution containing TBAHFP background electrolyte in the potential range from 0.0 V to 1.0 V wrt the saturated calomel electrode (|SCE|) (Author's own work).

#### 1.1.2 Information obtainable from electroanalytical methods

Electropolymerization can be followed by measuring successive CVs^13,14^ where an increase in currents shows the formation of the ECP on the electrode surface. Additionally, the polymer grown on the WE surface can be visually observed. Once the polymer is formed on the electrode surface, the electrode containing the polymer is removed from the polymerization solution, rinsed with the solvent, and CVs are recorded in the BGE without the monomer present in it. Removal of the monomer allows for the investigation of the redox properties of the polymer formed. Fig. 1(B) shows the CV recorded in the BGE of polyterthiophene grown on a Pt surface (Author's own work). As the potential is increased in the positive direction, say starting from 0 V, the polymer becomes electroactive at +0.639 V wrt SCE where the onset of the positive anodic current begins to appear. As the potential is increased, the current tends to increase and plateau. When the potential is reversed, a sudden decrease in current is typical behaviour of an ECP comparable to discharging a pseudo-capacitor. However, as the potential is swept in the reverse direction, the charge carriers are slowly removed, and the conductivity remains down to a certain potential beyond which it asymptotically reaches zero (Fig. 1(C)). As the scanning is continued in the negative direction, the polymer becomes n-doped due to the addition of electrons to the polymer and consequently, a negative current onset is reached beyond which negative current increases. In some cases, it is possible to observe redox peaks due to the presence of redox centres in the polymer. These peaks may correspond to polaron and bipolaron formation, as will be explained later. As the potential is swept in the positive direction, the oxidation potential onset,^15^*E*_(ox,onset)_, at which anodic current begins to appear, corresponds to the potential of the highest occupied molecular orbital (HOMO) level since the latter corresponds to the minimum energy required to remove an electron from the polymer. As the potential is swept in the negative direction, the reduction potential onset,^16^*E*_(red,onset)_, at which cathodic current begins to appear, corresponds to the potential of the lowest unoccupied molecular orbital (LUMO) level as the LUMO level is implicated by the minimum energy required to add an electron to the polymer. This is shown in Fig. 1(C). The HOMO and LUMO levels are generally expressed in terms of energy in electronvolts (eV) with respect to (wrt) the vacuum level where the energy of the vacuum is taken to be zero (Fig. 1(C)). Usually, the most used internal reference is the ferrocene/ferrocenium couple (Fc, Fc^+^) with a potential of −4.80 V wrt vacuum. The half-wave potential of Fc, Fc^+^ is +0.05 V wrt standard Ag(s)/AgCl(s)/KCl(sat. aq) electrode, where *E*_(1/2,Fc,Fc^+^)_ = [*E*_ap_(Fc, Fc^+^) + *E*_cp_(Fc, Fc^+^))]/2 = (0.01 V + 0.09 V)/2 = 0.05 V, where *E*_ap_ and *E*_cp_ are the anodic and cathodic peak potentials, respectively.^17^ Therefore, the HOMO and LUMO levels of the polymer can be expressed in energy terms wrt vacuum, respectively, by the eqn (1) and (2).^17^

1 *E*_HOMO_ = −[4.8 − *E*_1/2_(Fc, Fc^+^) + *E*_ox,onset_] = −(4.75 + *E*_ox,onset_)

2 *E*_LUMO_ = −[4.8 − *E*_1/2_(Fc, Fc^+^) + *E*_red,onset_] = −(4.75 + *E*_red,onset_)

**Scheme 1:**
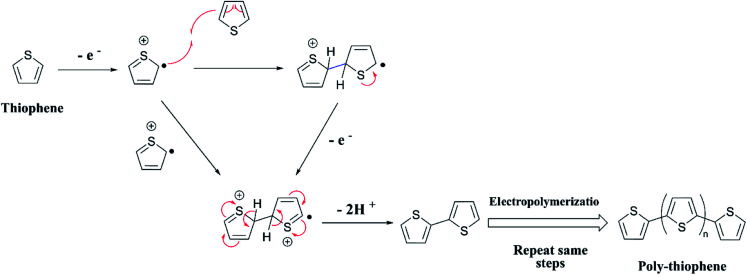
Mechanism of the polymerization of thiophene.

The two equations are very much like those empirically observed by Brédas *et al.*, for several EPCs with different chain lengths, but their equations use a different estimate of the relation between the electrochemical and vacuum energy scales leading to 4.50 instead of 4.75 eV. In any case, the HOMO–LUMO gap is the difference between the *E*_(red,onset)_ and *E*_(ox,onset)_.^17^ In the case of polyterthiophene, *E*_(ox,onset)_ = +0.639 V and *E*_(red,onset)_ = −0.769 V and hence *E*_HOMO_ = −5.389 eV and *E*_LUMO_ = −3.981 eV, thus giving rise to HOMO–LUMO gap (electrochemical band gap) of 1.408 eV. The UV-visible-NIR spectrum of polyterthiophene, shown in Fig. 1(D), has the absorption onset corresponding to the excitation of an electron from the HOMO level to the LUMO level at 653 nm (Author's own work). This corresponds to an optical HOMO–LUMO gap (optical band gap) of 1.899 eV. The electrochemical band gap is measured when the polymer deposited on the WE surface is in contact with the BGE. Therefore, electrolyte species and solvent molecules are incorporated into the polymer coat. The optical band gap is measured when the polymer coat deposited on the FTO surface is dry. Therefore, the molecular arrangements in the two states may be different and that may account for a small difference of 0.218 eV observed for the band gap of the polymer when measured by the two different methods. Additionally, the HOMO–LUMO gap for the hypothetical isolated gas-phase molecule of the polymer can be deduced from computer simulations. The HOMO–LUMO gap reported for polyterthiophene from computational simulations using TD-DFT/B3LYP 6-31G(d,p) is 2.420 eV.^18^ As an example for the mechanism of electropolymerization of monomers leading to ECPs, the mechanism of thiophene for the formation of polythiophene is shown in Scheme 1.

As the standard electrode potentials decrease and more monomer units are joined together,^19,20^ electropolymerization of a dimer (*e.g.*, bisthiophene, T_2_)^21^ can be carried out at a much lower potential than that of the monomer (thiophene, T). The electropolymerization of the trimer (terthiophene, T_3_)^22^ can be affected at even a lower potential than that required for the dimer. For instance, the potential onsets (wrt Ag|AgCl|KCl(sat'd) electrode) for the oxidative polymerization of T, T_2_ and T_3_ in the condensed phase in acetonitrile, as determined by Camarada *et al.*, are +1.50 V, +1.15 V and +0.880 V, respectively^23^ as shown in their Table 5, which is reproduced with permission in Table 1 here. The data show the electrochemical oxidation potentials and band gaps of thiophene oligomers in CH_2_Cl_2_ and CH_3_CN *versus* Ag|AgCl|KCl(sat'd)^24–26^ electrode determined using the thermochemical cycle with vertical ionization potential (IP), thermochemical cycle with adiabatic IP and the HOMO approximation in the gas phase and HOMO approximation in the condensed phase. The data show that the HOMO–LUMO gaps drastically decrease as the number of monomer units in the oligomer is increases. As shown in Fig. 1(D), there is a considerable absorption up to 1800 nm (MID-UV) with an absorption maximum of around 1600 nm in poly(T_3_) showing the optical HOMO–LUMO gap of 0.775 eV.

**Table 1:** Theoretical and experimental oxidation potentials (in V) in CH_2_Cl_2_ and CH_3_CN *versus* Ag|AgCl|KCl(sat'd) electrode using the thermochemical cycle with vertical IP, thermochemical cycle with adiabatic IP, HOMO approximation in the gas phase and HOMO approximation in the condensed phase of neutral (R) and radical cation (R^+^˙) species. This table has been reproduced from ref. 23 with permission from *Journal of Polymer Science B* (Polymer Physics) copyright 2011

Starting unit	*E*_R^+^˙|R_(CH_2_Cl_2_)					*E*_R^+^˙|R_(CH_2_CN)					Gap^a^
	[1]	[2]	[3]	[4]	Exp.	[1]	[2]	[3]	[4]	Exp.	
1st (T)	2.162	2.000	2.102	2.094	1.55	1.958	1.797	2.123	2.100	1.50	6.10
2nd (T_2_)	1.433	1.118	1.357	1.368	1.17	1.276	0.960	1.357	1.368	1.15	4.44
3rd (T_3_)	1.145	0.766	1.041	1.063	0.96	1.013	0.632	1.041	1.063	0.88	3.74
4th (T_4_)	0.980	0.593	0.915	0.853	0.90	0.864	0.475	0.827	0.853	0.82	3.25
5th (T_5_)	0.915	0.480	0.720	0.730	—	0.810	0.373	0.720	0.745	—	3.00
6th (T_6_)	0.903	0.397	0.652	0.660	0.85	0.807	0.299	0.652	0.674	0.85	2.83
MUE	0.239	0.291	0.207	0.216		0.161	0.326	0.239	0.242		

a HOMO–LUMO gap in eV in gas and condensed phase.

#### 1.1.3 Charge carriers in electronically conducting polymers

**Fig. 2:**
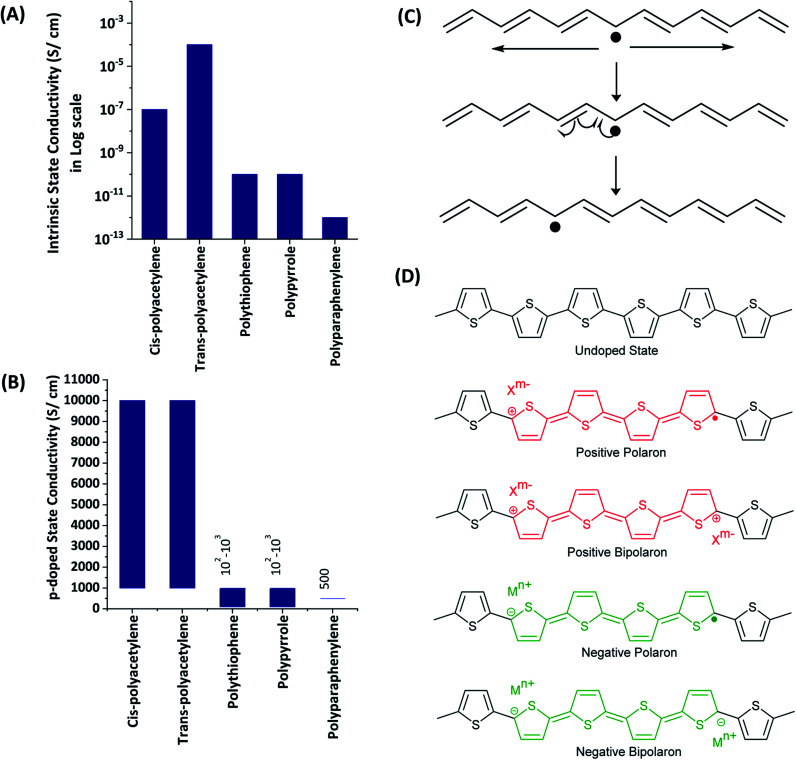
Conductivities of some electronically conducting polymers in the (A) intrinsic and, (B) p-doped states. (C) Illustration of the native solitons of *trans*-polyacetylene. (D) Charge carriers such as positive, negative polarons and positive, negative bipolarons of polythiophene.

All the ECPs have the common characteristics of extended conjugation in the polymer backbone. This extended conjugated structure provides the path for electrons or holes to move along the polymer backbone. However, the extended conjugated path itself is not sufficient for the material to possess electronic conductivity owing to either bond alternation (Peierls distortion) or electron–electron interaction effects. There should be charge carriers. Most of the common ECPs have very low or negligible intrinsic electronic conductivity as shown in the data given in Fig. 2(A). The significant conductivity of *trans*-polyacetylene is due to the presence of intrinsic solitons (extended conjugated free radicals), as shown in Fig. 2(B). The conductivity of the ECPs can be increased by deliberately introducing charge carriers by the doping process. This can be carried out by using chemical oxidants/reductants or electrochemically by applying sufficient positive or negative potentials. The electrochemical doping of the ECPs to possess the necessary p- or n-type conductivity is easily achieved by subjecting the polymer grown on the electrode surface to the required potential ranges. The applied potential can also control the extent of doping and consequent conductivities. The charge carriers present in EPCs are positive or negative polarons (extendedly conjugated cation or anion radicals) and positive or negative bipolarons (extendedly conjugated positive or negative charges on two atoms which are formed by the combination of two respective polarons) as shown in Fig. 2(C) for polythiophene. The presence of charges in the polymer backbone requires counter ions to balance the overall charge to zero. As such, when the polymer is oxidized to possess positive charges, anions from the solution are incorporated into the structure. When the backbone contains negative charges, cations are inserted. The presence of two charged layers separated by trapped electrolyte explains the electrical double layer and consequent pseudo-capacitance.

#### 1.1.4 Developmental stages of electronically conducting polymers

Shirakawa, Louis, MacDiarmid, Chiang and Heeger published the first paper on ECPs titled “Synthesis of Electrically Conducting Organic Polymers: Halogen Derivatives of Polyacetylene, (CH)_*x*_”, in 1977, where they claimed that new field of chemistry was born.^27^ Shirakawa was working on the polymerization mechanism of acetylene using Ziegler–Natta catalysts. Heeger was working on the physics of quasi-one-dimensional conductor materials such as TTF–TCNQ (tetrathiafulvalene-7,7,8,8-tetra-*p*-quinodimethane). At the same time, McDiarmid was working on sulphur nitride (SN)_*x*_ conducting inorganic polymers. Chiang used molar quantities of the Zieglar–Natta catalyst for acetylene polymerization and obtained an intriguing product having metallic lustre. Together, the team of scientists showed that the new material they synthesized had high electronic conductivity and it could be enhanced drastically by exposing to iodine vapor. This was the discovery that led a path to develop electronically conducting organic polymers. Since then, the field of ECPs has grown exponentially and several generations of ECPs have been discovered. The original discoverers of ECPs were honoured by the award of the Nobel Prize in Chemistry-2000.^28–30^ In the 50^th^ anniversary perspective of conducting/semiconducting conjugated polymers, Swager describes his perspective on the past and the future of ECPs where he describes an evolutionary chain of ECPs in which (SN)_*x*_ polymers are claimed to be the starting materials of the evolutionary chain.^31^ Polyacetylene and other carbon-based homo ECPs are the 1^st^ generation of ECPs. Then, those with heteroatoms such as S, N, O in conjugated carbon chains is considered the 2^nd^ generation of ECPs. The third generation of ECPs are comprised of D–A type polymers. In this review, we discuss the developments in D–A type ECPs synthesized from 2004 to 2021 *via* electropolymerization. The D–A type ECPs are important for many reasons: (i) they possess considerably high intrinsic electronic conductivity when compared to other types of ECPs, (ii) their optical absorption and emission extend towards the near to middle infrared region and hence they stand out as excellent bio-markers and bio-imaging materials, (iii) they show excellent electrochromic properties due to fast, stable and intense colour changes upon changing applied potential, (iv) they can be made soluble in common solvents when monomers used have solubilizing side-chains and the polymers demonstrate solvatochromic properties, (v) they are widely used in optoelectronic devices such as organic solar cells and the proper choice of D and A materials enabling high efficiencies for solar energy-to-electricity conversion and (vi) they are used as high-performance materials in field-effect transistors.

**Scheme 2:**
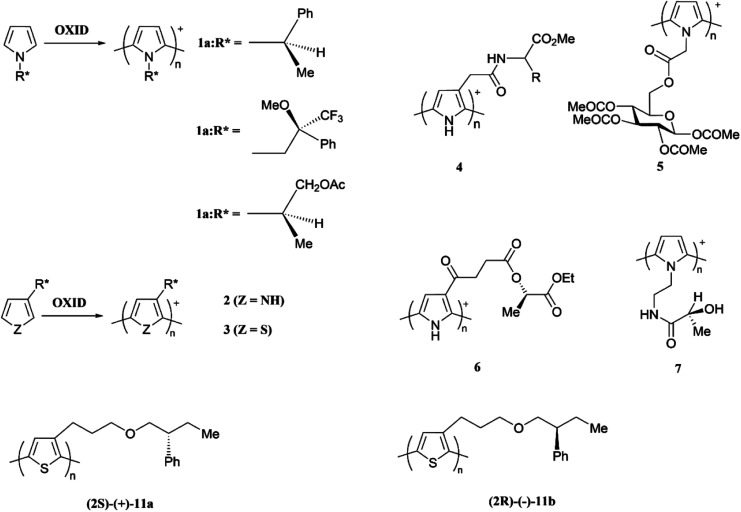
Chirality of some electronically conducting polymers. This figure has been reproduced from ref. 32 with open access permission from RSC Chemical Society Reviews to *RSC Journals*, copyright 2010.

#### 1.1.5 Chirality in electronically conducting polymers

The term chirality describes the configuration or handedness (left or right) of an optically active chemical compound containing asymmetric carbon atom(s); a carbon atom with four different groups attached to it. The compound and its mirror image are non-superimposable and are called enantiomers. One compound rotates the plane of the plane polarized light in one direction, say, clockwise and the other rotates the plane of the plane polarized light in the other direction (anti-clockwise). An equimolar mixture of the two enantiomers is called a racemic mixture and is optically inactive. The chirality is often observed in nature where essential biomolecules such as the natural amino acids and simple sugars are often chiral and preferentially exist in only one enantiomeric form. Biomolecules such as proteins, nucleic acids and DNA have helical chirality contributing to the exceptional selectivity in metabolic processes in biological species where only one type of enantiomer binds to the active site of enzymes since the enzyme catalysis often depends on the size and shape of the active site. As such, in the development of pharmaceutical products chiral separation is essential since one enantiomer is an active drug while other is not. In the recent years, the chirality of ECPs has been emerged as a very important concept and chiral ECPs have been developed. These materials find application in electrochemical chiral sensing and in electrochemical asymmetric synthesis.^32^ Monomers of pyrrole and thiophene with chiral groups attached to the heteroatom have ben polymerized to have ECPs with chiral pendant groups. Some examples are given below with relevant references. There are excellent reviews on chiral ECPs^33^ and readers are directed to them since the elaborative discussion of chiral ECPs is beyond the scope of this review (Scheme 2).^34–40^

## 2. Electropolymerization of mixtures of monomers

### 2.1 Electronically conducting block copolymers of controlled stoichiometry

**Fig. 3:**
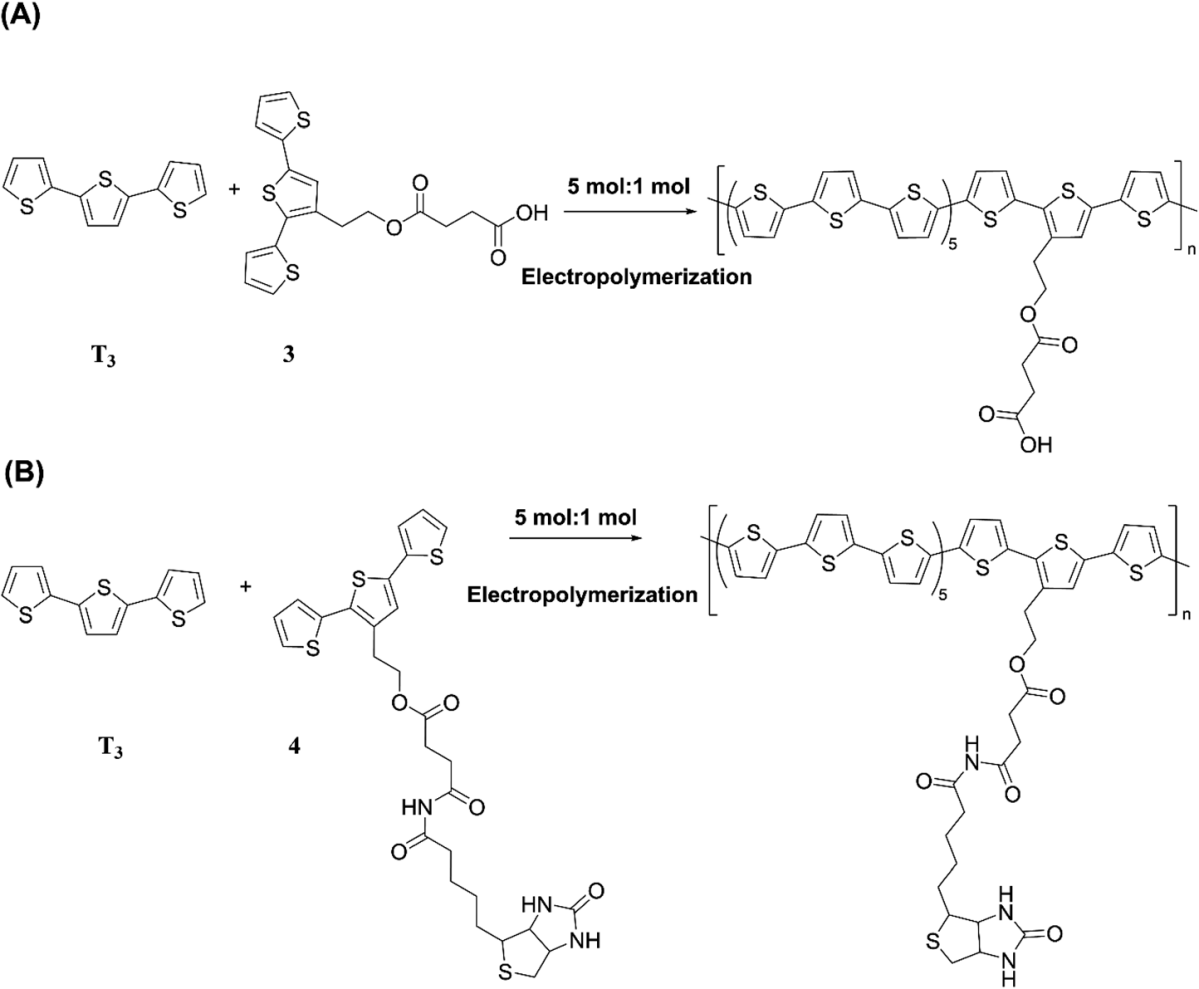
(A) Reaction scheme showing the synthesis of 2-(2,5-dibromo-3-thienyl)ethanol (1), 2-[2,2′:5′,2′′]-terthiophen-3′-yl-ethanol (2), succinic acid mono-(2-[2,2′:5′,2′′] terthiophene-3′-yl-ethyl)ester (3) and (4-{*N*′-[5-(4,6-dioxo-hexahydro-thieno[3,4-*c*]pyrrol-3-yl)-pentanoyl]-hydrazino}-4-oxo)-butyric acid 2-[2,2′:5′,2′′]terthiophene-3′-yl-ethyl ester (4). (B) Electropolymerization of 5 : 1 molar ratio of 2,2′:5′,2′-terthiophene (T_3_) and 3 or 4 leading to the formation of electronically conducting copolymers, poly[(T_3_)5-3] or poly[(T_3_)5-4] with the same stoichiometry in the monomer blocks.

The versatility of electropolymerization can be extended to synthesize electronically conducting copolymers also. First attempted by Mouffouk *et al.* in 2004,^41^ the approach of using an easily oxidizable monomer with one that is difficult to oxidize for electropolymerization leads to electronically conducting copolymers. In a consecutive synthesis scheme, they prepared 2-(2,5-dibromo-3-thienyl)ethanol (compound 1) starting from 2-(3-thienyl)ethanol and allowing it to react with *N*-bromosuccinimide in dry tetrahydrofuran. Compound 1 was then converted to 2-[2,2′:5′,2′′]-terthiophen-3′-yl-ethanol (compound 2). Compound 2 was converted to succinic acid mono-(2-[2,2′:5′,2′′]terthiophene-3′-yl-ethyl) ester (3) and starting with 3 they prepared (4-{*N*′-[5-(4,6-dioxo-hexahydro-thieno[3,4-*c*]pyrrol-3-yl)-pentanoyl]-hydrazino}-4-oxo)-butyric acid 2-[2,2′:5′,2′′]terthiophene-3′-yl-ethyl ester (4) as shown in Fig. 3(A). They have then electropolymerized 3 and 4 separately with 2,2′:5′,2′′-terthiophene (T_3_) in 5 : 1 molar ratio in each case, to result in the respective copolymers (Fig. 3(B)). Here, the easily oxidizable T_3_ monomer acts as the polymerization initiator and coupling agent to aid the insertion of complex monomers (either 3 or 4) in the copolymer chain together with T_3_ as one monomer repeat unit. They also showed that the molar ratios of the two monomers present in the block copolymer could be conveniently controlled by the molar ratio of the two respective monomers taken for the polymerization. As such, 5 : 1 molar ratio each of T_3_ and monomers 3 or 4 gave the same molar ratio of the repeat units in the block copolymers, (T_3_–T_3_–T_3_–T_3_–T_3_–T_3_-3)_*n*_ and (T_3_–T_3_–T_3_–T_3_–T_3_–T_3_-4)_*n*_, respectively, as determined by the elemental analysis. This observation also shows the power of electropolymerization in controlling the stoichiometry of the copolymers. Interestingly, the successive CVs in the synthesis of poly(T_3_), poly[(T_3_)5-3] and poly[(T_3_)5-4] have some similarities and distinct differences. They have shown that *E*_ox,onset_ values of T_3_, T_3_ and 3 in 5 : 1 molar ratio, and T_3_ and 4 in 5 : 1 molar ratio are the same at +0.650 V wrt SCE, that corresponds to the *E*_ox,onset_ of T_3_ indicating that T_3_ to be the polymerization initiator in each case. This result is further supported by the fact that both 3 and 4 on their own without T_3_ is unable to electropolymerize to yield their respective homopolymers. However, the redox properties of the three polymers are distinctly different, particularly in the anodic scan. Further, the rates of polymerization, implied by the magnitudes of the currents generated, follow the order *T*_3_ > T_3_ and 3 in 5 : 1 molar ratio > T_3_ and 4 in 5 : 1 molar ratio. Structurally, the only difference is that in every sixth T_3_ unit contains bulky groups in 3 and 4 where 4 contains much bulkier group than that in 3. The incorporation of bulky groups in the polymer tends to increase steric hindrance for the access of the counter ions from the solution, thereby lowering the rate of polymerization.

**Fig. 4:**
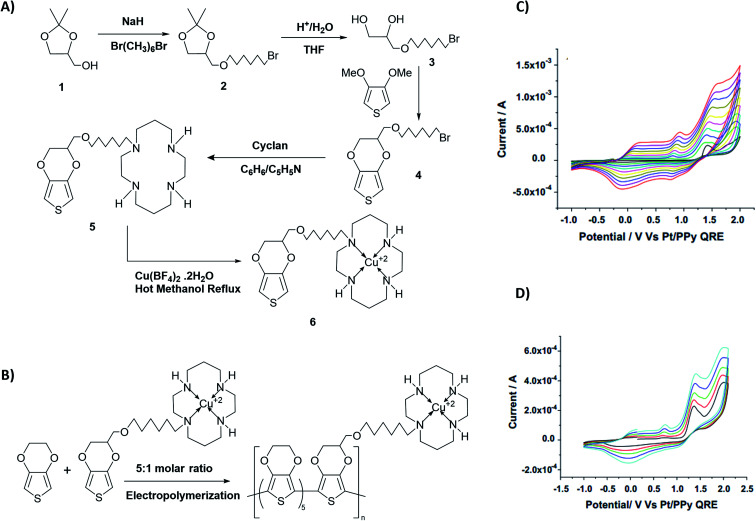
The synthesis reaction scheme of {(6-((2,3-dihydrothieno[3,4-*b*][1,4]dioxin-2-yl)methoxy)hexyl)1,4,8,11-tetraazacyclotetradecane} copper(ii) tetrafluoroborate [Cu(5)(BF_4_)_2_] (compound 6). (B) Compound 6 electropolymerization reaction with EDOT. (C) The repetitive scan CVs for the polymerization reaction, and (D) the repetitive scan CVs for the polymerization of EDOT under the same conditions. This figure has been reproduced from ref. 45 with permission from *Electrochimica Acta*, copyright 2010.

From the respective CVs of (i) poly(T_3_), (ii) poly[(T_3_)5-3] and (iii) poly[(T_3_)5-4] in the same background electrolyte at scan rates of (1) 10, (2) 20, (3) 30 and (4) 40 mV s^−1^, the researchers have also shown that the electroactivity of the three polymers follow the same order, poly(T_3_) > poly[(T_3_)5-3] > poly[(T_3_)5-4] because the anodic currents generated follow the same order. Additionally, when the polymers are discharged by switching the potential scan to the negative direction, the potential onset values at which the cathodic currents reach zero also follows the order poly(T_3_) > poly[(T_3_)5-3] > poly[(T_3_)5-4] showing that the discharging of the doped polymers also becomes difficult when they contain bulky groups even if they are present attached only to every 6th T unit. Discharging of the polymer requires the expelling of the counter ions from the polymer into the solution, and the presence of bulky groups tends to slow down this process also due to steric hindrance.^42^ Consequently, the electronic conductivities of the three polymers, measured using AC impedance analysis, at different applied DC potentials,^43,44^ also follow the same order when the polymers are in their p-type states. In addition to investigating fundamental properties of homo- and co-polymers based on T and chemically modified T, the researchers have also shown that the simple electropolymerization leading to ECPs containing functionalized moieties such as biotin groups can be used as electrochemical sensors to detect important biological species such as streptavidin targets. Velauthamurthy *et al.* modified EDOT to have a 1,4,8,11-tetraazacyclotetradecane (cyclam) ligand attached to one of the carbon atoms of the ethylenedioxy bridge of the EDOT through methoxyhexyl spacer group to result in 6-(2,3-dihydrothieno[3,4-*b*][1,4]dioxin-2-yl)methoxy)hexyl)-1,4,8,11-tetraazacyclotetradecane (compound 5) by chemical modification of EDOT as shown below. They have also prepared a Cu(ii) coordinated by the cyclam ligand of compound 5 by allowing to react compound 5 with [Cu(BF_4_)_2_·2H_2_O], by refluxing in methanol and synthesized {(6-((2,3-dihydrothieno[3,4-*b*][1,4]dioxin-2-yl)methoxy)hexyl)1,4,8,11-tetraazacyclotetradecane} copper(ii) tetrafluoroborate [Cu(5)(BF_4_)_2_] (compound 6). Monomer 6 was copolymerized with EDOT in the molar ratio of EDOT to monomer in the range 4 or 5 to result in the copolymers with the same molar ratio.^45^Fig. 4 shows (A) the synthesis reaction scheme, (B) electropolymerization reaction, (C) the repetitive scan CVs for the polymerization reaction, and (D) the repetitive scan CVs for the polymerization of EDOT under the same conditions. They have found that the co-polymerization method affords good control of the metal concentration in the polymer matrix and represents a good technique for preparing electronically conductive polymers containing redox-active metal complexes. On the first scan of the polymerization of EDOT and 6 in 5 : 1 molar ratio (in the positive direction), three prominent anodic peaks appear at +0.94 V, +1.35 V and +1.60 V, respectively, wrt Pt/PPy quasi-reference electrode (Fig. 4(C)). These peaks are assigned to the polaron and bipolaron formation and further oxidation of bipolaron. The corresponding values of PEDOT are +0.75 V, +1.25 V and +1.82 V, respectively (Fig. 4(D)).

**Scheme 3:**
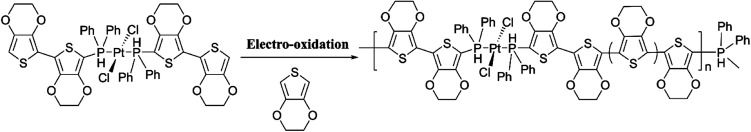
Electropolymerization of diphenyl(2,20,3,30-tetrahydro-[5,50-bithieno[3,4-*b*][1,4]dioxin]-7-yl)phosphane (Ph2P[bis-EDOT]) complex (7) with ethylenedioxythiophene in *x* : 1 molar ratio to yield the copolymer with the same numbers of monomer units in the blocks. This figure has been reproduced from ref. 48 with open access permission from ScienceDirect, copyright 2017.

Comparison of the data shows that polarons and bipolarons form at higher potentials in the copolymer and those in the PEDOT indicating the effect of the bulky side chain on the redox properties of PEDOT even if the bulky side chain is attached in every 6th EDOT unit. This again shows the effect of steric hindrance on redox properties of EPCs. Also, bipolarons formed in PEDOT are more stable at high potentials than those present in the copolymer^46,47^ since further oxidation of bipolarons in PEDOT demands a higher potential. Interestingly, the first CV of copolymer formation has a perfectly reversible redox couple at −0.30 V wrt Pt/PPy quasi-reference electrode and is absent in the CV of the PEDOT. This behaviour is due to the redox behaviour of Cu(ii)/Cu(i) couple since Cu(ii) is present coordinated to the cyclam ligand in the copolymer. This reversible redox peak is masked due to high currents of the polymer in the subsequent CVs. Velauthamurthy *et al.* have also worked on the synthesis and characterization of [MCl_2_(PAr_3_)_2_] – ethylenedioxythiophene copolymers (M = Pd, Pt), made by electropolymerization of diphenyl(2,2′,3,3′-tetrahydro-[5,5′-bithieno [3,4-*b*][1,4]dioxin]-7-yl phosphane (Ph2P[bis-EDOT]) complex (7) with EDOT as the polymerization initiator, coupling agent and a monomer (Scheme 3). The resulted polymer structure is shown in Scheme 3. It has been shown that the use of *x* : 1 molar ratio of the EDOT and monomer 7 gave the same molar ratio in the blocks of the copolymer.^48^ The conventional ECPs usually do not have appreciable intrinsic conductivity,^49^ but they gain high conductivities upon p- or n-doping. The electrochemical impedance analysis at various DC bias potential values in both positive and negative regions (for potentials (*e.g.*, from −1.0 V to +1.0 V in 0.10 V intervals) can be used to determine both electron transfer resistance, Ret, and the accompanying ion-transport resistance, RI. These polymers have very large resistance values in the potential range between *E*_ox,onset_ and *E*_red,onset_ which show that they behave like electronic insulators in their intrinsic states (Fig. 1(C)). However, they have very small Ret and RI values at potentials more positive than *E*_(ox,onset)_ (due to p-doping) and more negative than *E*_(red,onset)_ due to n-doping. As the potentials increase in either direction, the resistance values decrease due to the formation of more charge carriers. Interestingly, the electrochemical impedance data of the copolymer of EDOT and 6 given in Table 1 (ref. 45) show considerably small resistance values in the range from −0.5 V to +0.5 V through 0 V. This data shows that this copolymer has intrinsic electronic conductivity. Also, the copolymer of EDOT and 7 show such intrinsic conductivity. This conductivity is due to electron-rich T_3_ and EDOT units acting as electron donors and electron-deficient moieties acting as acceptors.

### 2.2 Electropolymerization leading to donor–acceptor type of electronically conducting polymers

**Scheme 4:**
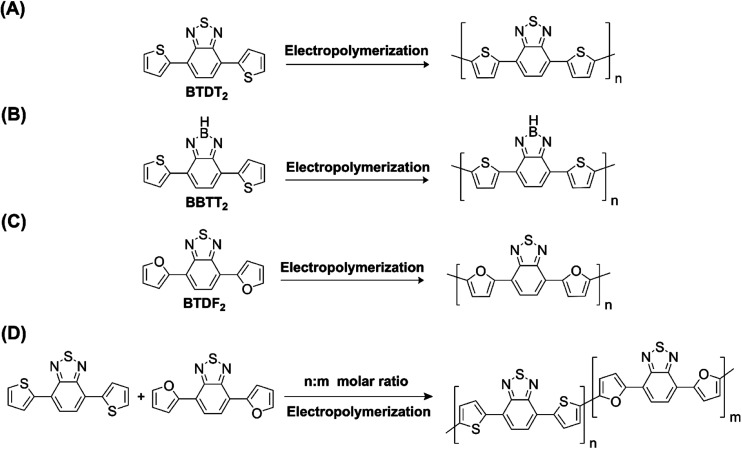
Electropolymerization reactions of (A) 4,7-dithiophen-2-yl-2,1,3-benzothiadiazole (BTDT_2_), (B) 2,5-di(thien-2-yl)-benzodiazaborole (BBTT_2_) (C) 4,7-difuran-2-yl-2,1,3-benzothiadiazole (BTDF_2_) and, (D) BTDT_2_ and BTDF_2_ mixtures in various molar ratios such as 1 : 1, 1 : 2, 2 : 1, 2 : 5 and 5 : 2 *etc.* This figure has been reproduced from ref. 42 with permission from RSC *Journal of Material Chemistry C*, copyright 2019.

**Fig. 5:**
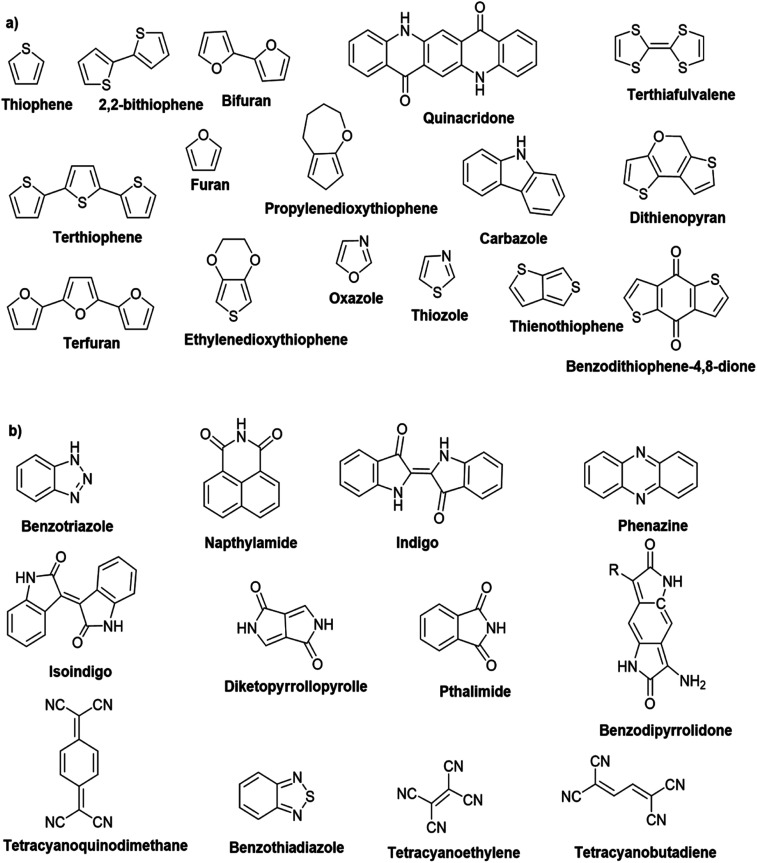
Commonly used (a) electron donors (D) and, (b) electron acceptors (A) in making D–A type polymers.

Hayashi and Koizumi^50^ synthesized 4,7-di(thien-2-yl)-2,1,3-benzothiadiazole (BTDT_2_) and 2,5-di(thien-2-yl)-benzodiazaborole (BBTT_2_) and electropolymerized, separately, to result in corresponding polymers. The structures of these monomers and the electropolymerization reactions are shown in Scheme 4. Successive CVs recorded during the polymerization show the increase in currents which indicates the formation of ECPs on the WE surface, in both cases. Although, BTD alone cannot be electropolymerized within the potential range chosen, the attachment of T groups to BTD or BBT enables the BTDT_2_ or BBTT_2_ monomer to polymerize electrochemically to result in (BTDT_2_)_*n*_ or (BBTT_2_)_*n*_ polymers. The CVs of the polymers recorded in the range from −1.5 V to +1.0 V wrt Ag(s)/AgCl(s)/KCl(aq, sat.) have measurable currents in the entire domain though the currents between *E*_ox,onset_ and *E*_red,onset_ are considerably lower than those outside this region. They have shown that the intrinsic conductivity within *E*_ox,onset_ and *E*_red,onset_ is due to donor–acceptor mechanism where electron-rich T units act as donors and electron-deficient BTD and BBT act as acceptors. Interestingly, the visible-NIR spectra of the monomer BBTT_2_ and the polymer (BBTT_2_)_*n*_ have clear differences. The band corresponding to the π → π* transition of the monomer seems to appear below 400 nm in the near UV region though it has been red shifted to around 420 nm in the polymer.^51,52^ The broad absorption band of the monomer cantered at 530 nm tails off to zero before 700 nm and there is no measurable absorption in the NIR region. However, the same band in the polymer has been red shifted to 680 nm in the polymer and it tails off to zero around 880 nm indicating that the polymer absorbs in the NIR region also. This is due to the decreasing in energy gaps due to the extendedly conjugated polymer backbone when compared to those of the monomer. Extending the concept of electropolymerization of complex monomers with those that are easily polymerizable to form copolymers, Rajapakse *et al.* polymerized 4,7-dithiophen-2-yl-2,1,3-benzothiadiazole (BTDT_2_) and 4,7-difuran-2-yl-2,1,3-benzothiadiazole (BTDF_2_) separately to form their respective homopolymers. Additionally, they electropolymerized BTDT_2_ and BTDF_2_ mixtures in various molar ratios such as 1 : 1, 1 : 2, 2 : 1, 2 : 5, 5 : 2 *etc.* leading to alternating and block copolymers where the stoichiometry of the blocks of the two monomers in the polymer was shown to be determined by the molar ratios of the two monomers taken for electropolymerization. They have shown that 1 : 1 stoichiometry of the two monomers gives alternating copolymer of the type –(A–B)_*n*_– while other stoichiometries give the block copolymers with the same number of respective monomer units in the blocks of the copolymer.^42^ This chemistry was proven by the CVs of the respective polymers as well as by the elemental analysis based on SEM-EDX. Here, the T or F units act as electron donors and BTD units act as electron acceptors making the polymers to have intrinsic electronic conductivities as confirmed by their EIS data. The EIS data show that poly(T_3_) is conducting only when p- or n-doped at more positive potentials than *E*_ox,onset_, and more negative potentials than *E*_red,onset_ respectively, of the polymer and between these potentials it behaves as an electrical insulator. However, (T-BTD-T)_*n*_ and (F-BTD-F)_*n*_ and all the copolymers of BTDT_2_ and BTDF_2_ show electrical conductivity throughout the potential range investigated (from −1.2 V to +1.2 V measured in 100 mV steps) though, at more positive potentials than *E*_ox,onset_, and more negative potentials than *E*_red,onset_, they show higher conductivities due to doping compared to intrinsic conductivities shown between this range. Nevertheless, the intrinsic conductivities of the copolymers are significantly higher than those of common ECPs. In these copolymers, the electron-rich moieties such as T, F, oligothiophenes, EDOT, *etc.* act as electron donors. The electron-deficient monomers joined in the polymer act as electron acceptors thus making them donor–acceptor (D–A)-type of EPCs if the energy levels match for donor moieties to transfer electrons to the acceptor moieties. This interaction results in the creation of the charge carriers in the intrinsic state thus making the D–A type polymers intrinsically electronically conducting. The donor molecules used in making D–A type polymers often contain electron-rich heteroatoms, such as sulphur, oxygen, and sp^3^-hybridized nitrogen. Popular donor molecules contain thiophene- or furan-based structures. Fig. 5(a) shows some donor molecules commonly used in making D–A type of polymers. The acceptor molecules used in making D–A type of polymers have electron-deficient aromatic rings or double bonds due to the presence of electron-withdrawing atoms or groups such as sp^2^ hybridized N, lactam groups (cyclic amide), C=N *etc.* Some of the commonly used acceptor molecules are shown in Fig. 5(b).

Table 3 presents data from 2005 to the present from the literature for D–A type polymers. The wealth of examples given in Table 3 shows that thiophene and its derivatives, including cyclopentadithiophene-4-one, dithienopyrrole, thieno[3,2-*b*]thiophene, bithiophenes, terthiophenes, disilanobithiophene, dithienogermolo dithiophene, EDOT, dithienopyran, 2,5-bis-dithienyl-1*H*-pyrrole are the most used D type of monomers. Additionally, conjugated carbon-based monomers such as phenylvinylenes and fluorene, and those containing sp^2^ N such as carbazole have been used as D molecules. Common A molecules used are quinoxaline, BTD, perylene diamide and diketopyrrolopyrrole (DPP) in making D–A type ECPs.

However, as the information in Table 2 shows, several other materials can act as D or A type of moieties. Suitable D and A combinations must be determined by evaluating the HOMO levels of D molecules and LUMO levels of A molecules experimentally CV and UV-visible-NIR spectroscopy^53^ or theoretically by computational method.

**Table 2:** The literature on D–A type polymers prepared from 2005 to the present with the demonstrated applications

Donor	Accepter	Polymer	Applications
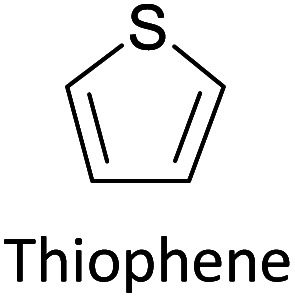	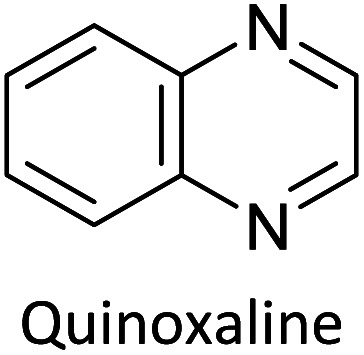	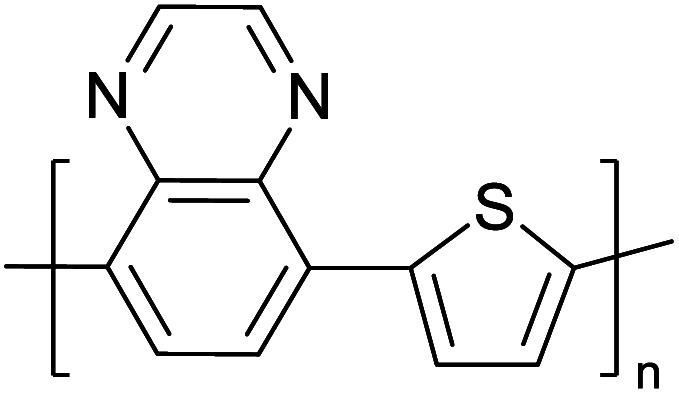	Electrochemical studies for future use^54^
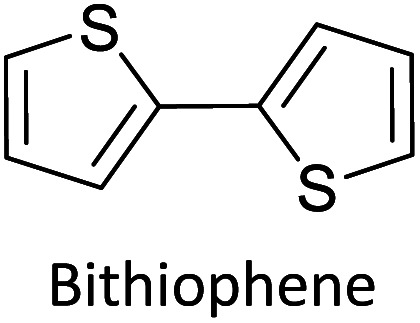	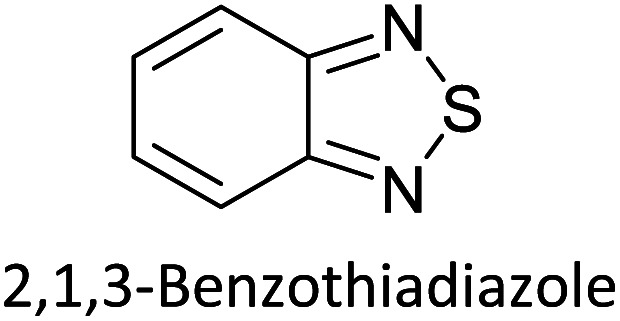	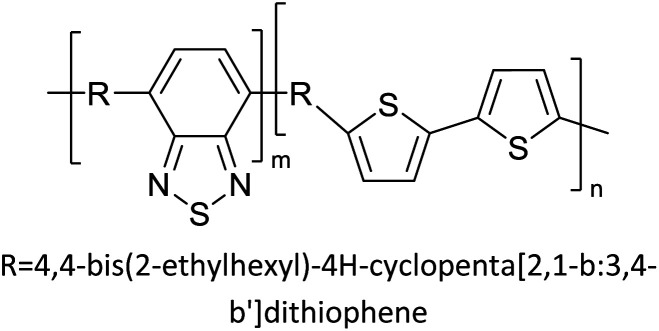	Organic solar cells^55^
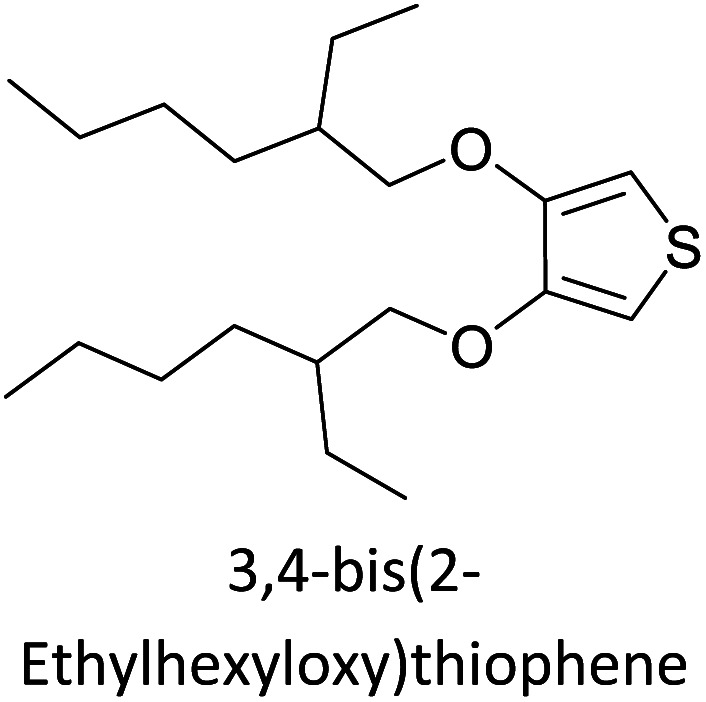	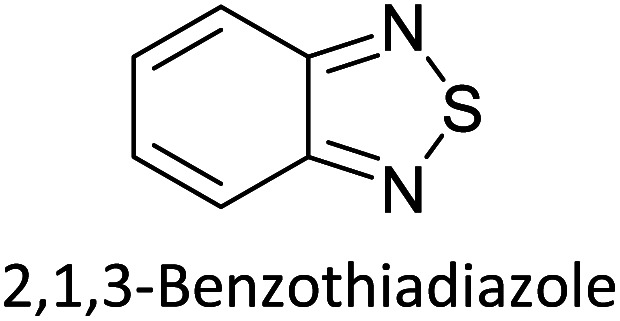	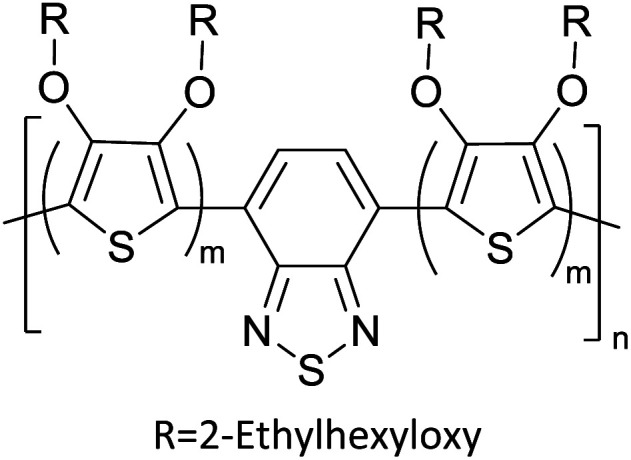	Heterojunction polymer solar cells^56^
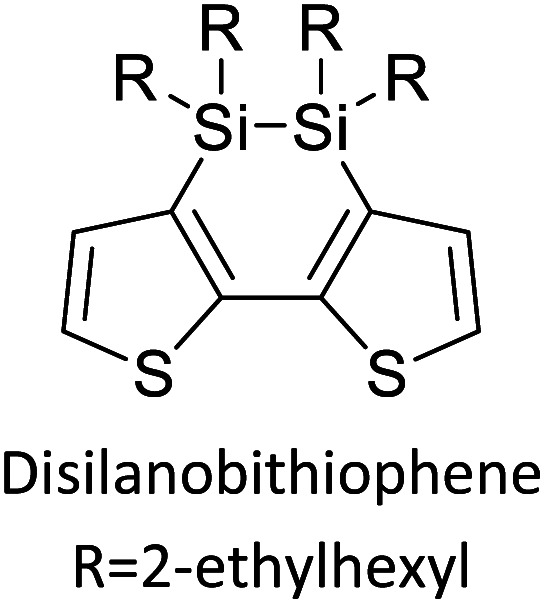	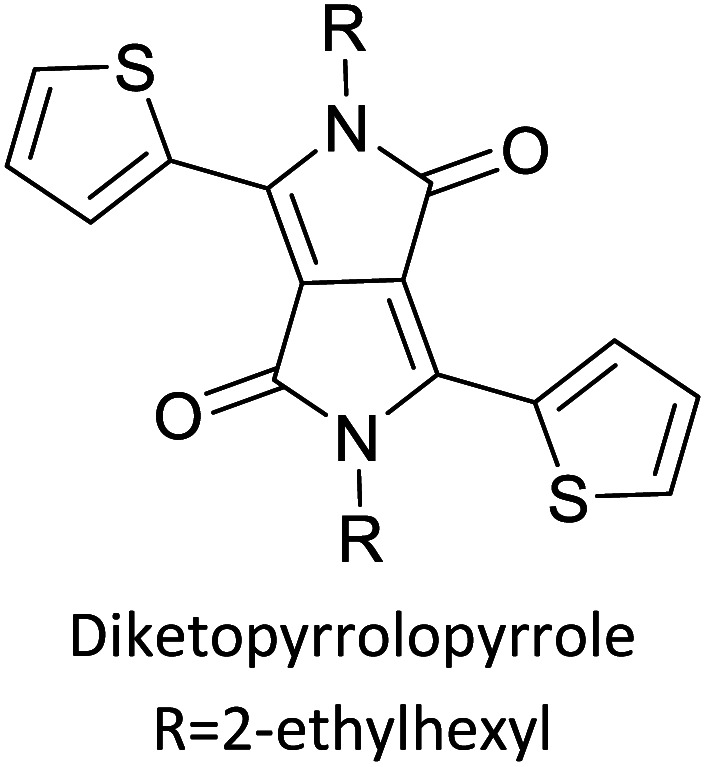	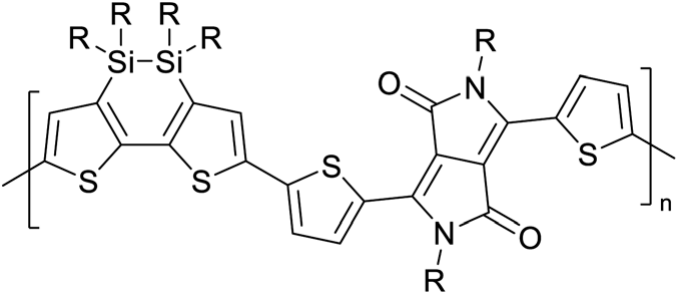	Heterojunction polymer solar cells^57^
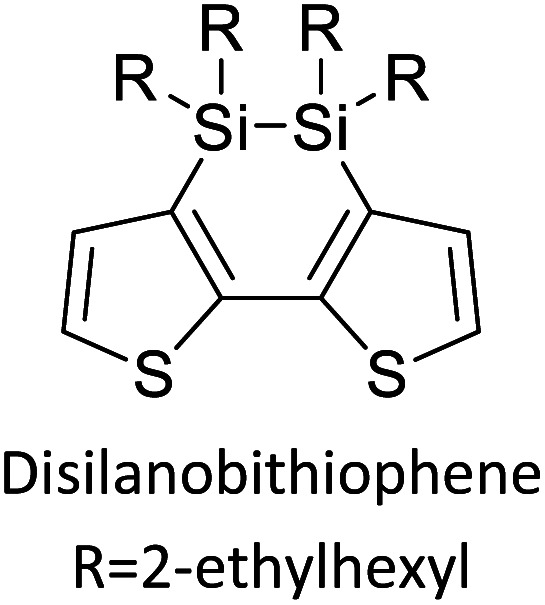	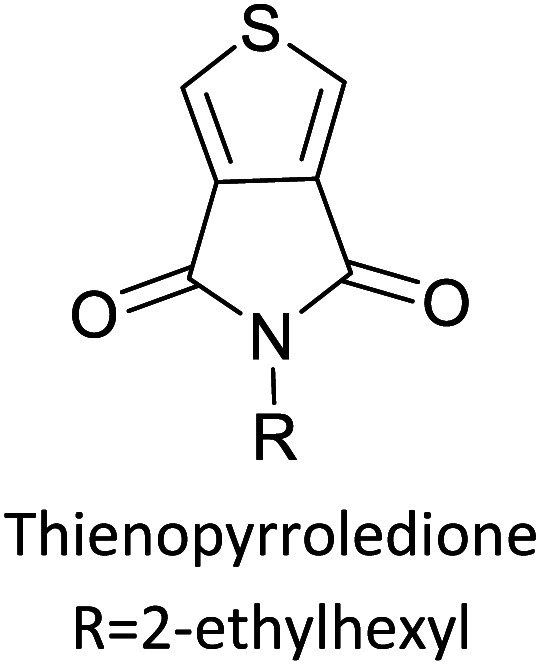	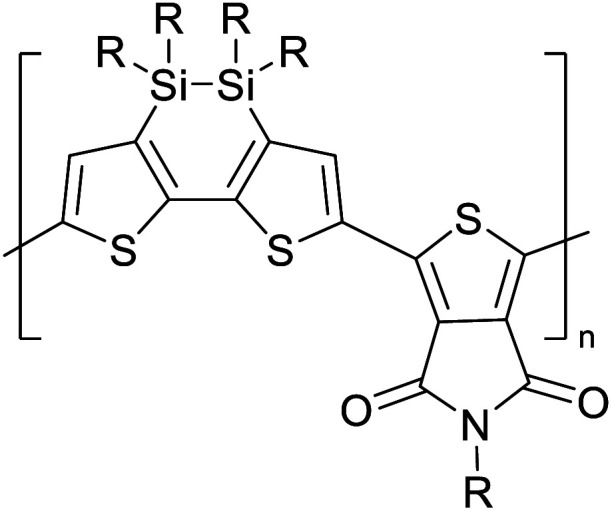	Bulk heterojunction polymer solar cells^57^
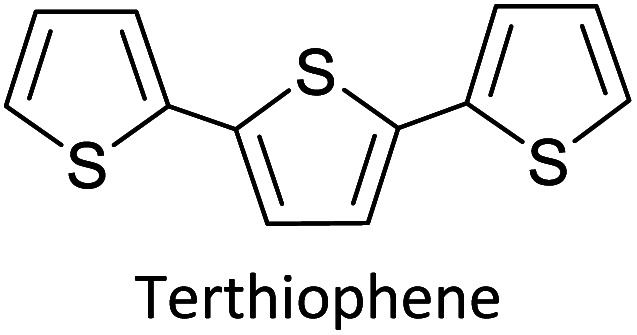	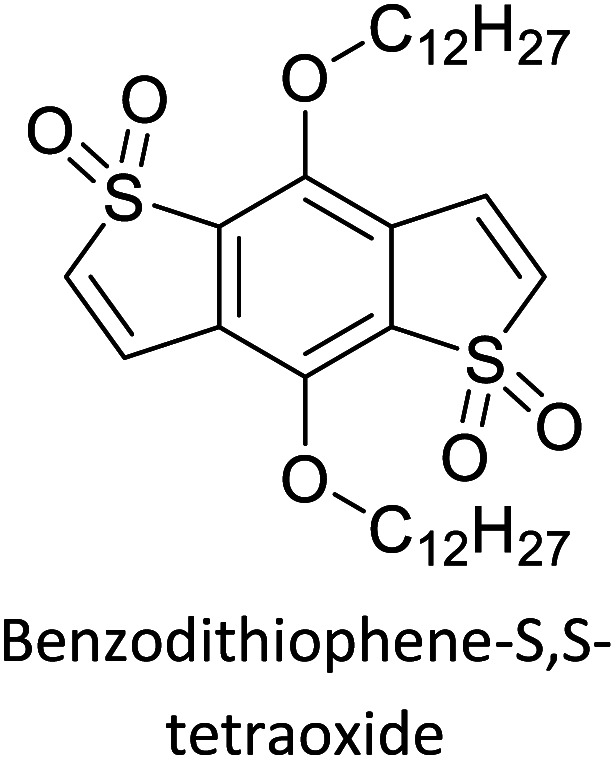	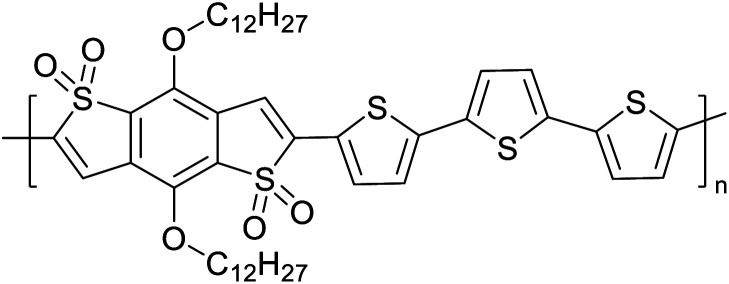	Semiconducting electropolymers^58^
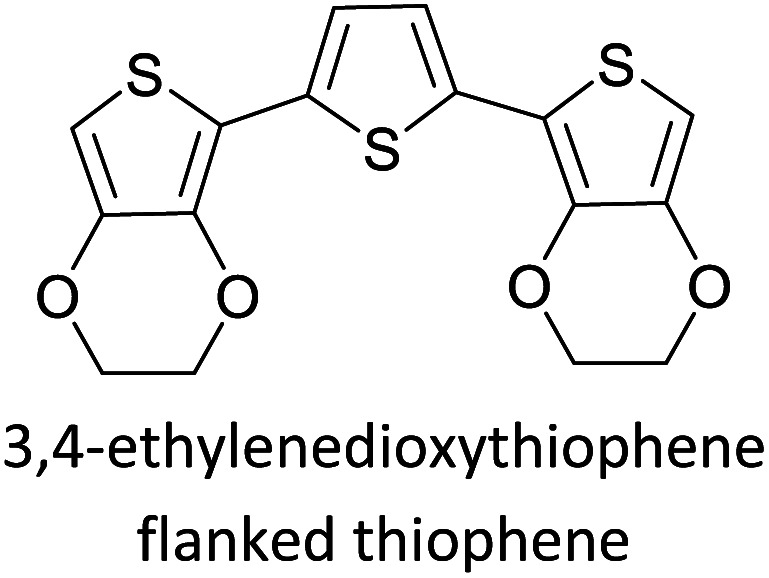	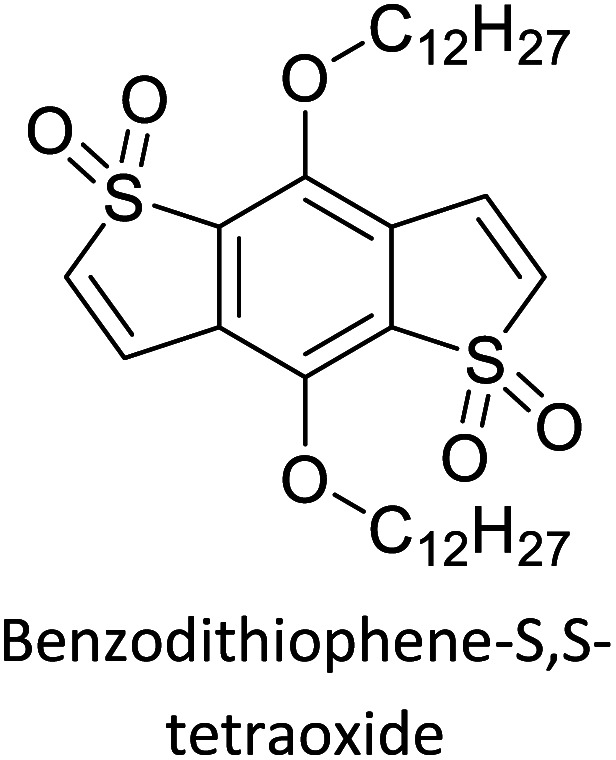	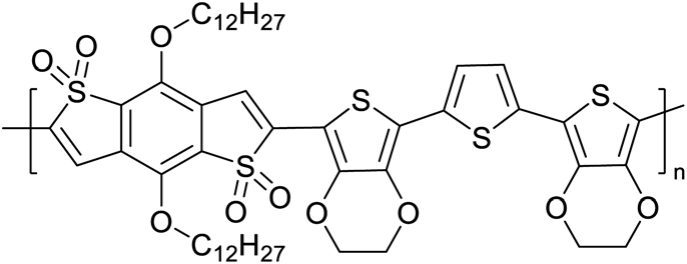	Semiconducting electropolymers^58^
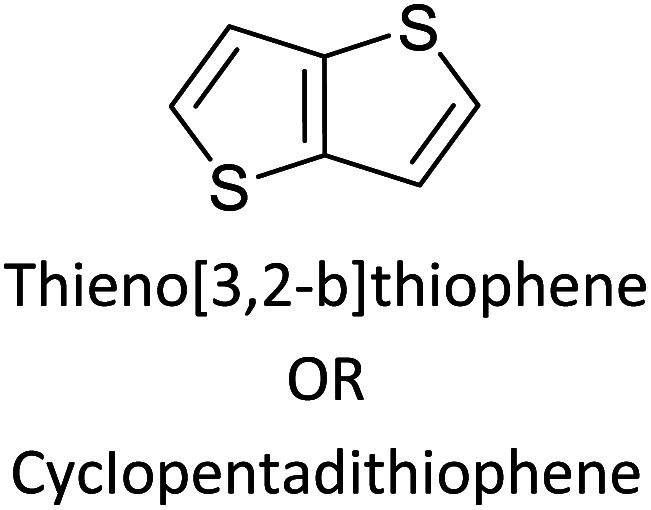	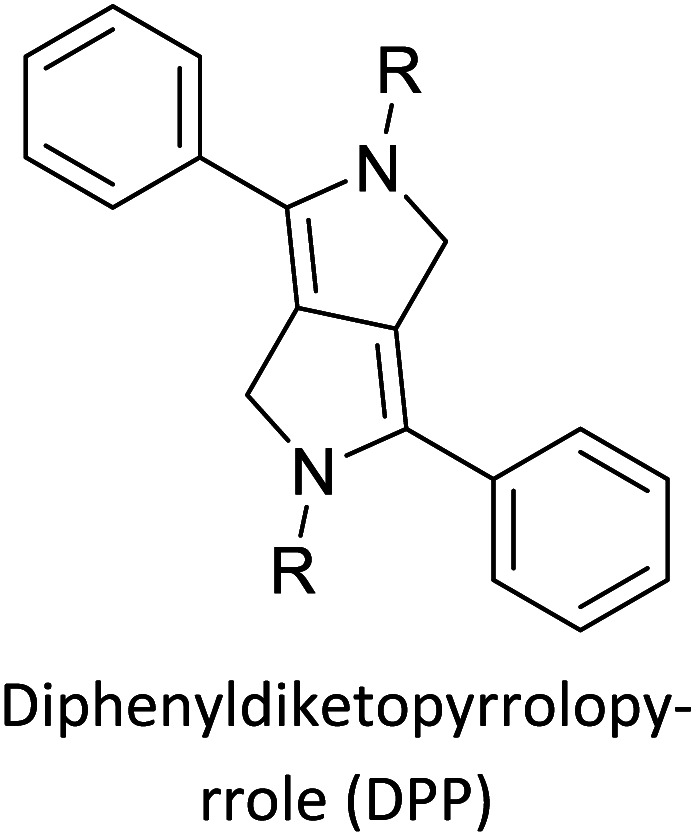	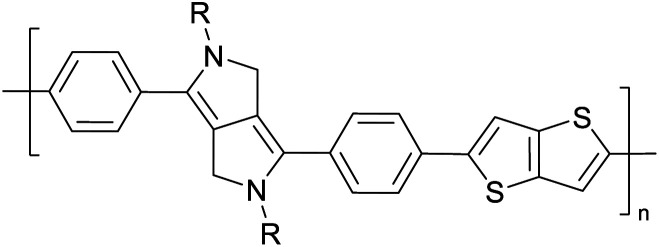	Development of semiconducting DPP-containing polymers for transistors^59^
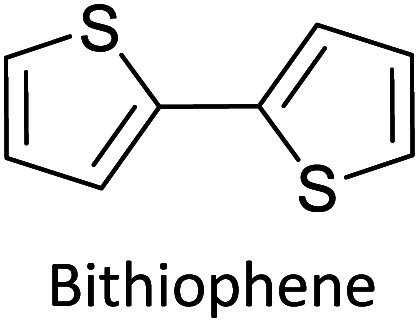	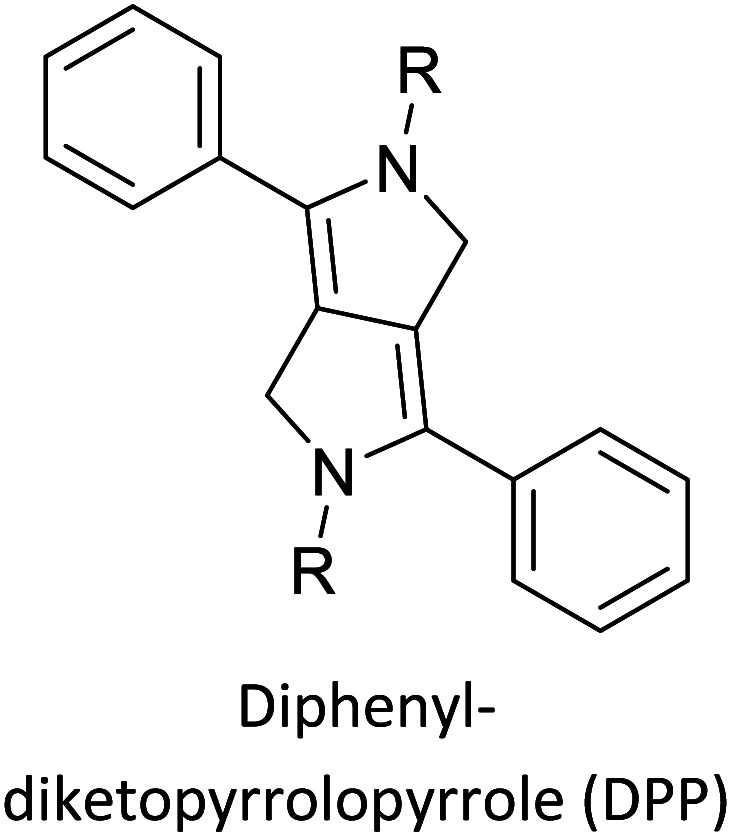	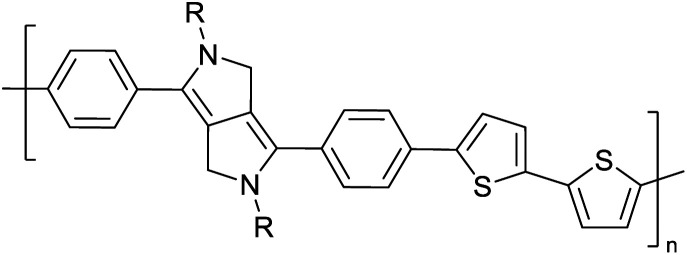	Development of semiconducting DPP-containing polymers for transistors^59^
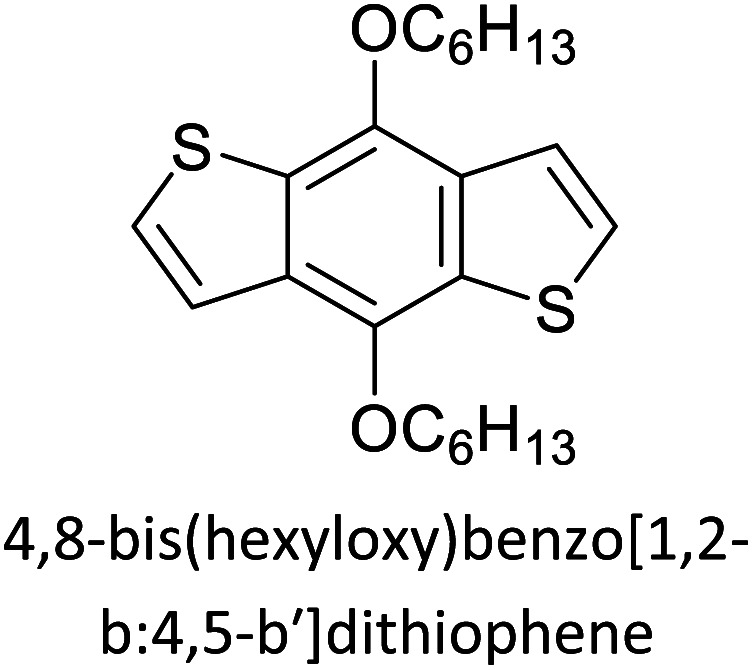	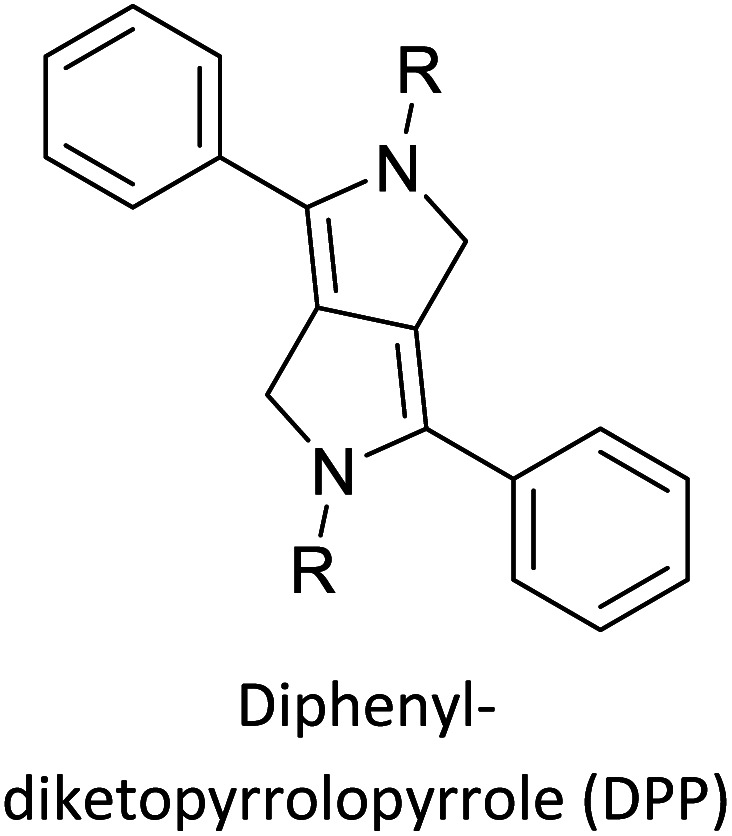	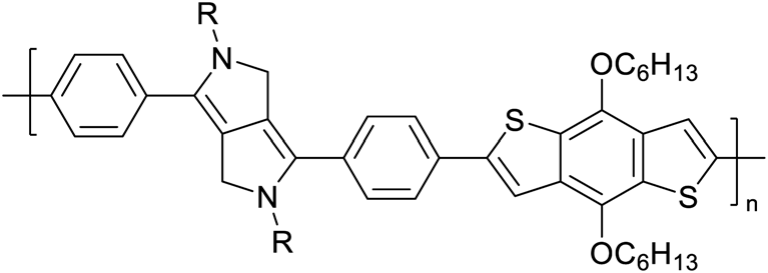	Development of semiconducting DPP-containing polymers for transistors^59^
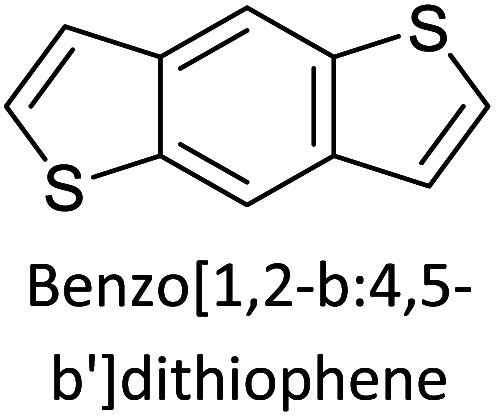	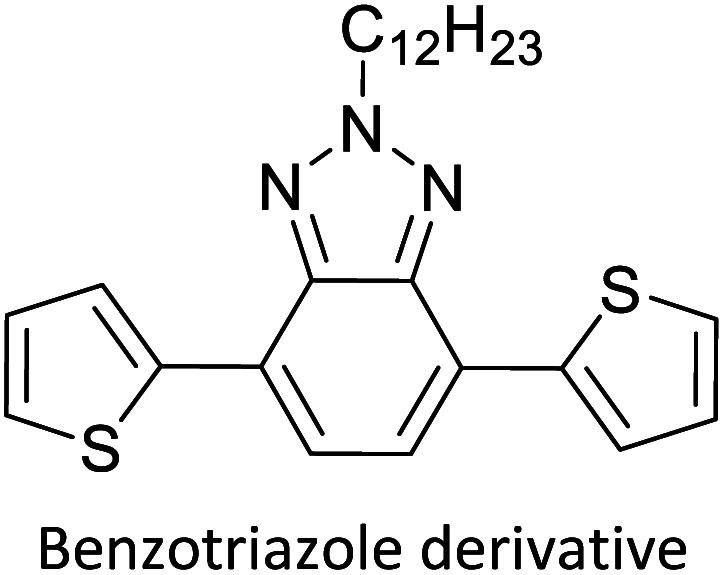	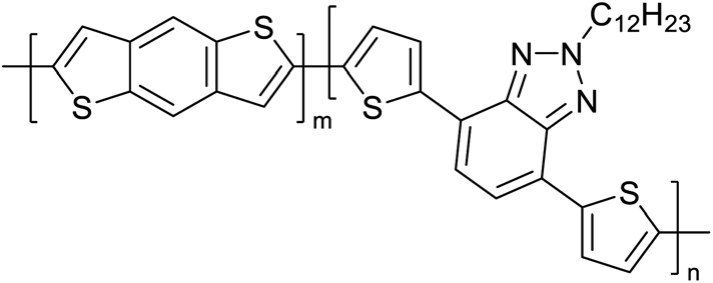	Electrochromic materials for commercial applications^60^
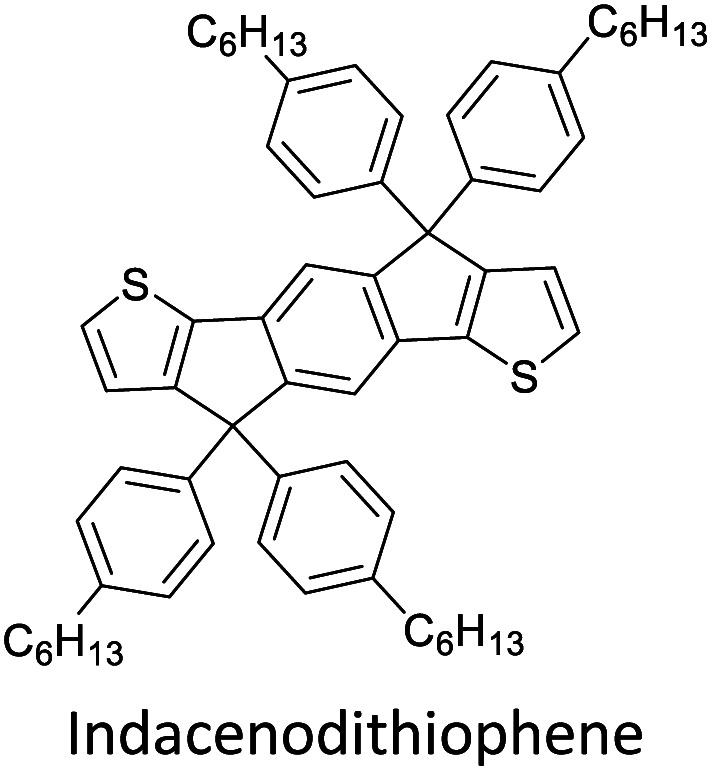	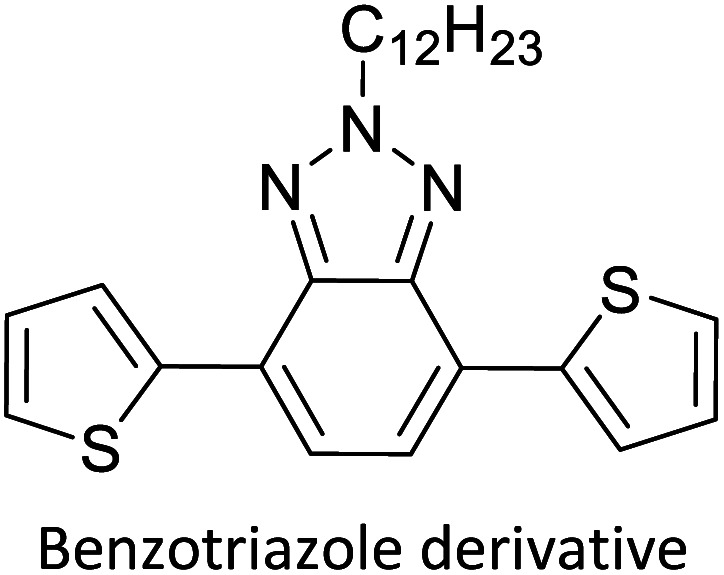	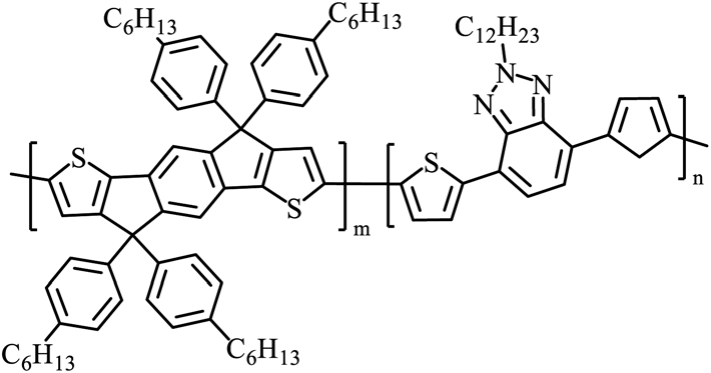	Electrochromic materials for commercial applications^60^
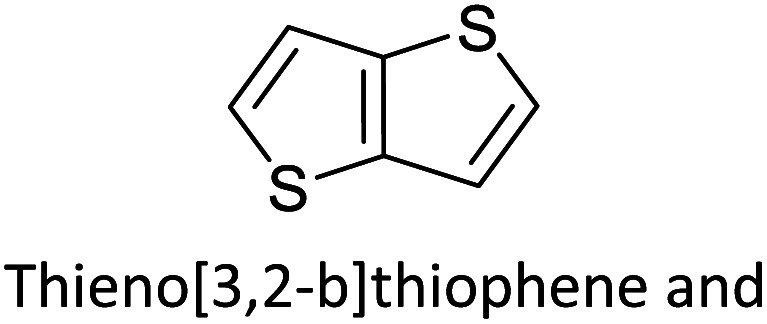	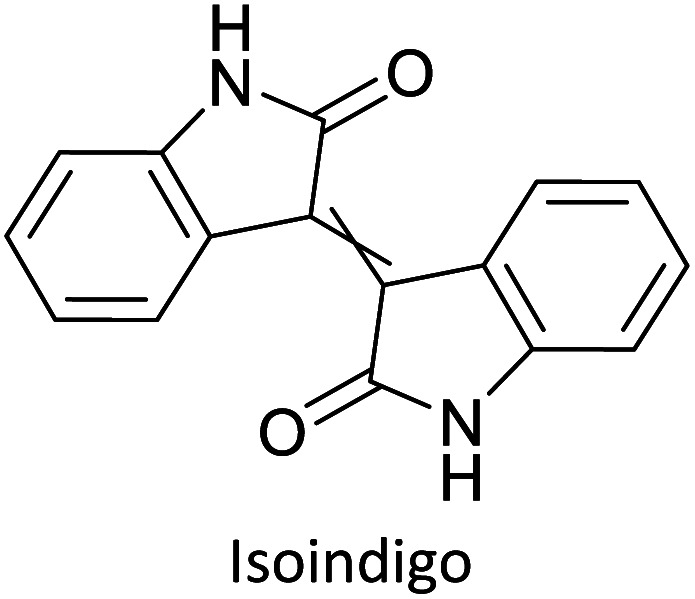	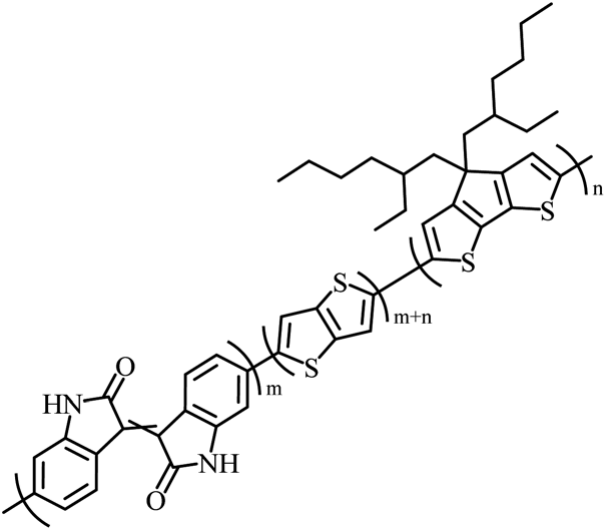	Electrochromic materials^61^
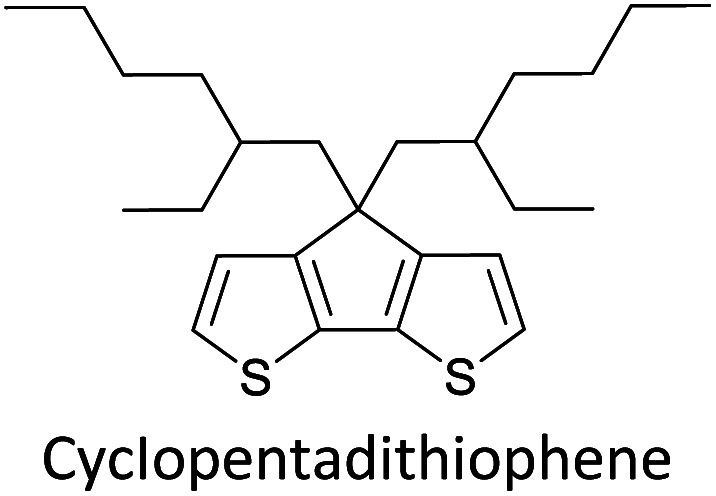			
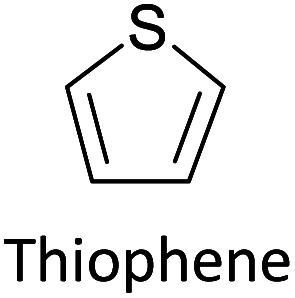	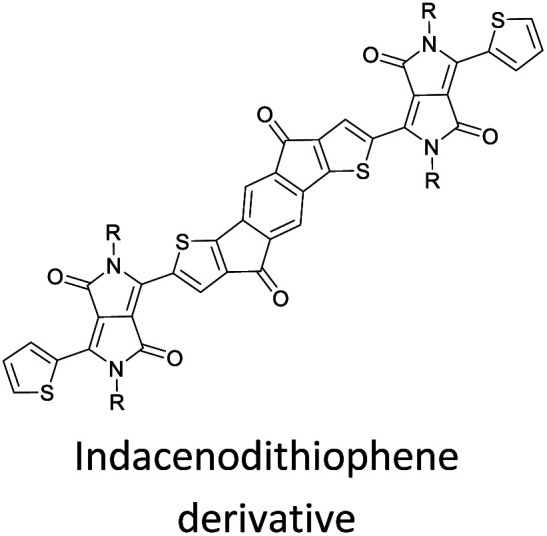	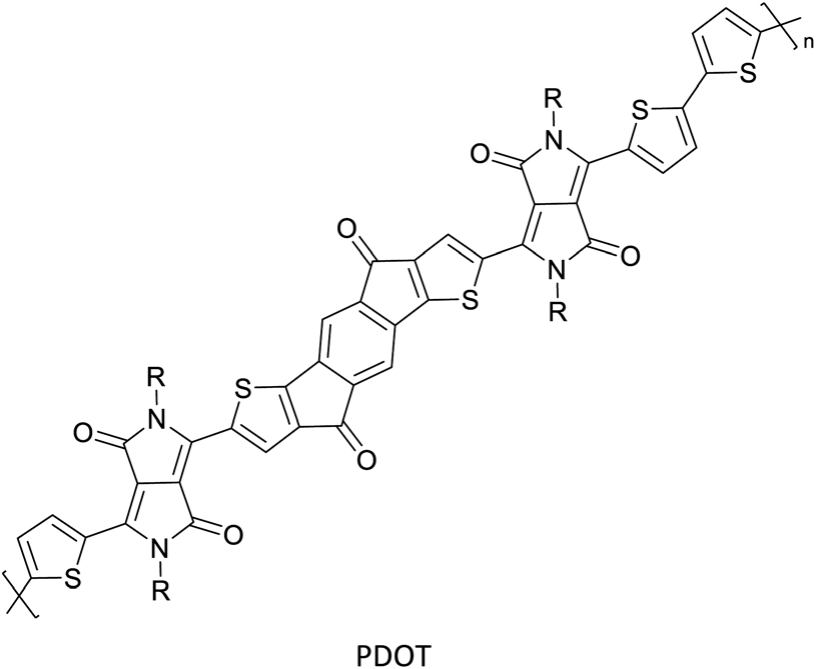	Semiconductors in optoelectronic devices^62^
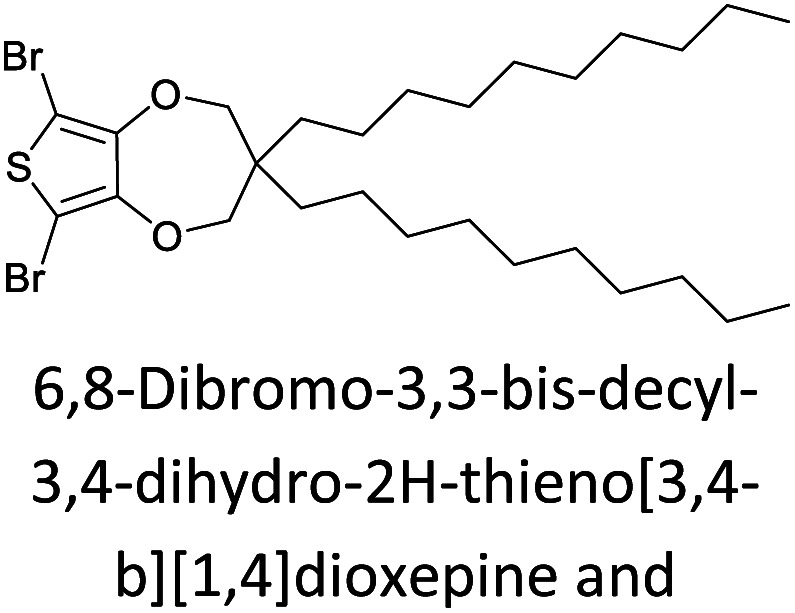	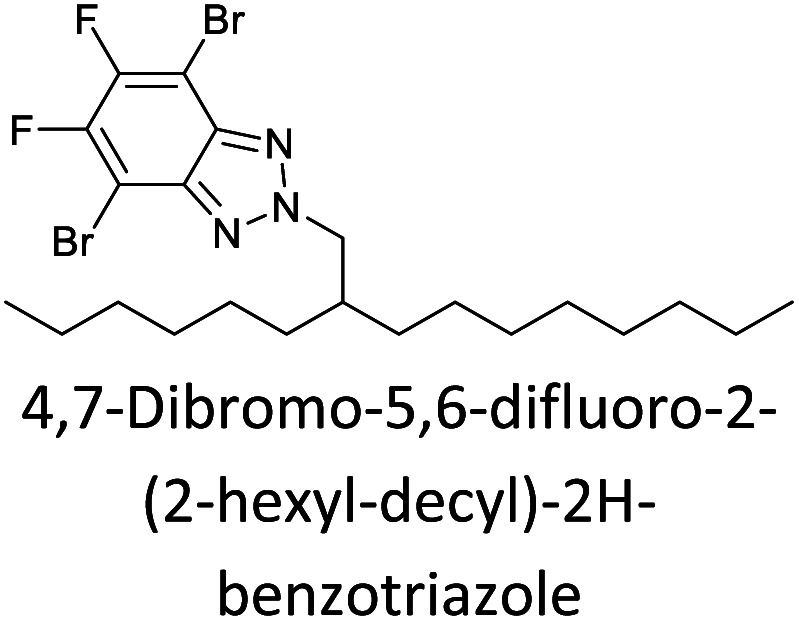	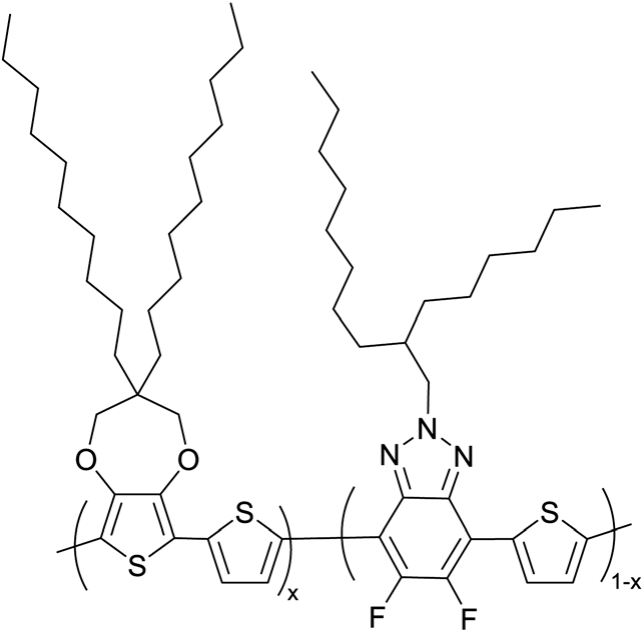	Electrochromic material^63^
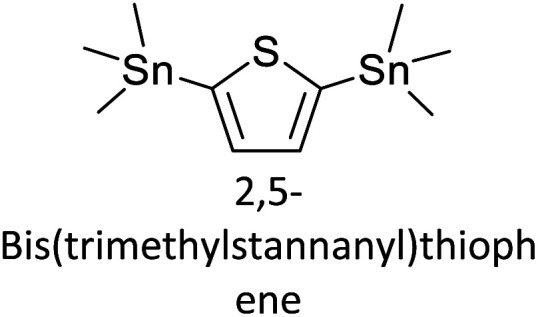			
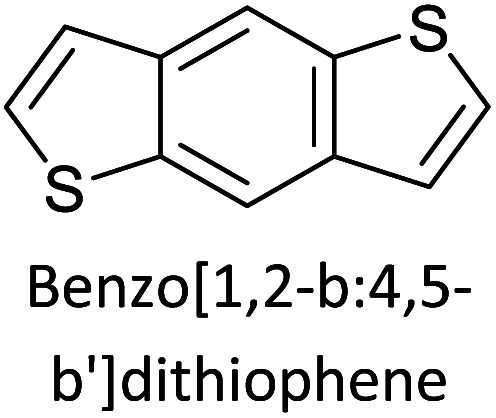	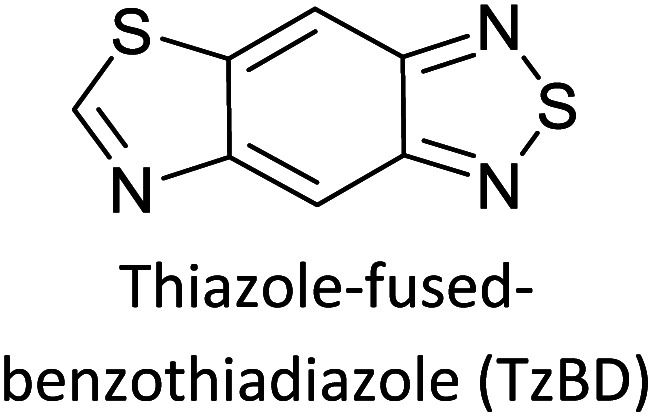	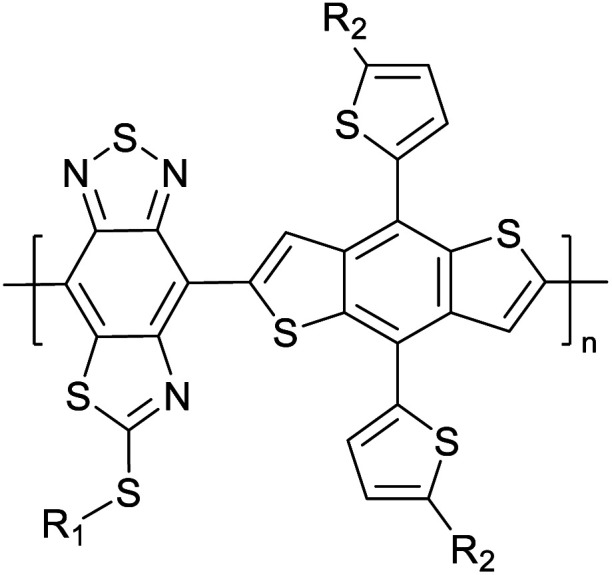	Organic solar cells^64^
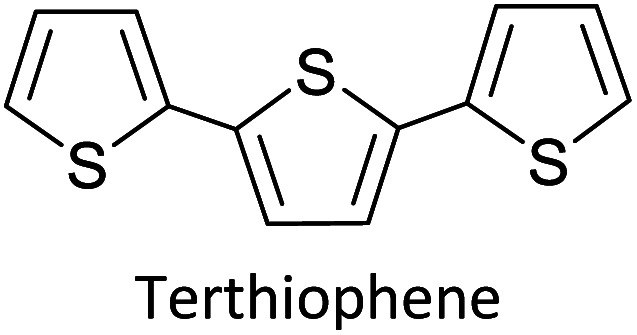	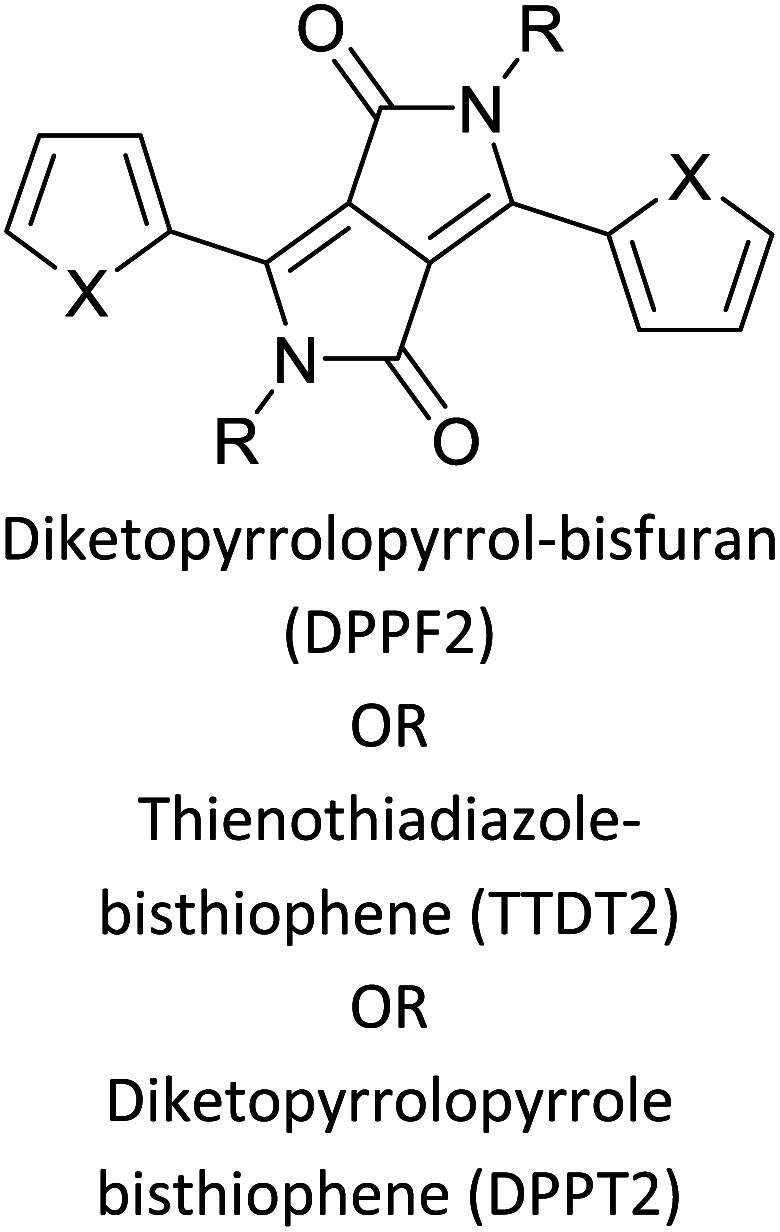	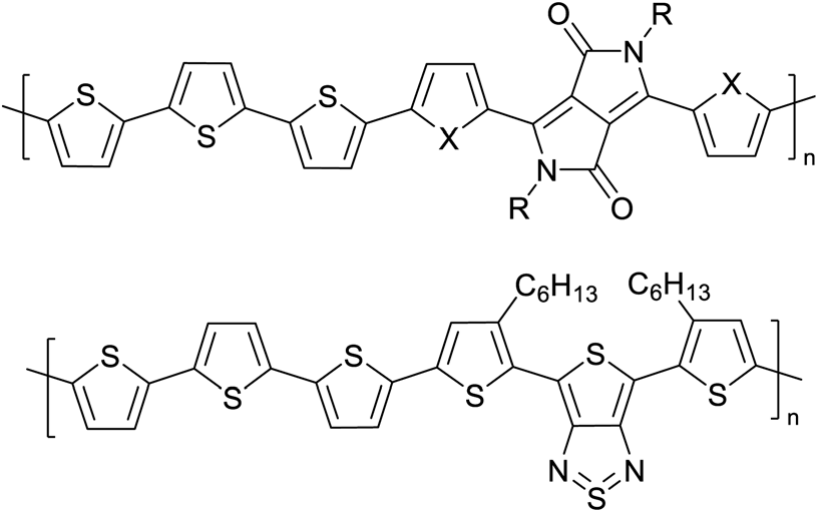	Optoelectronics^65^
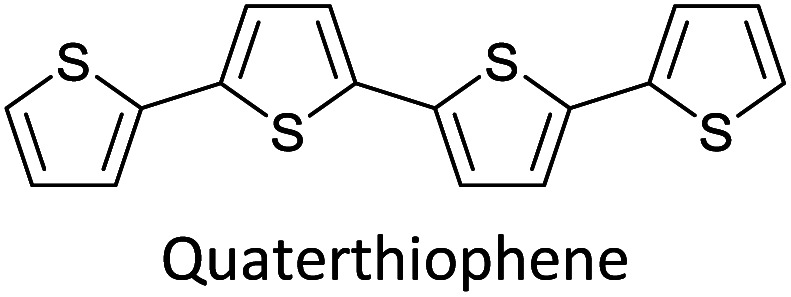	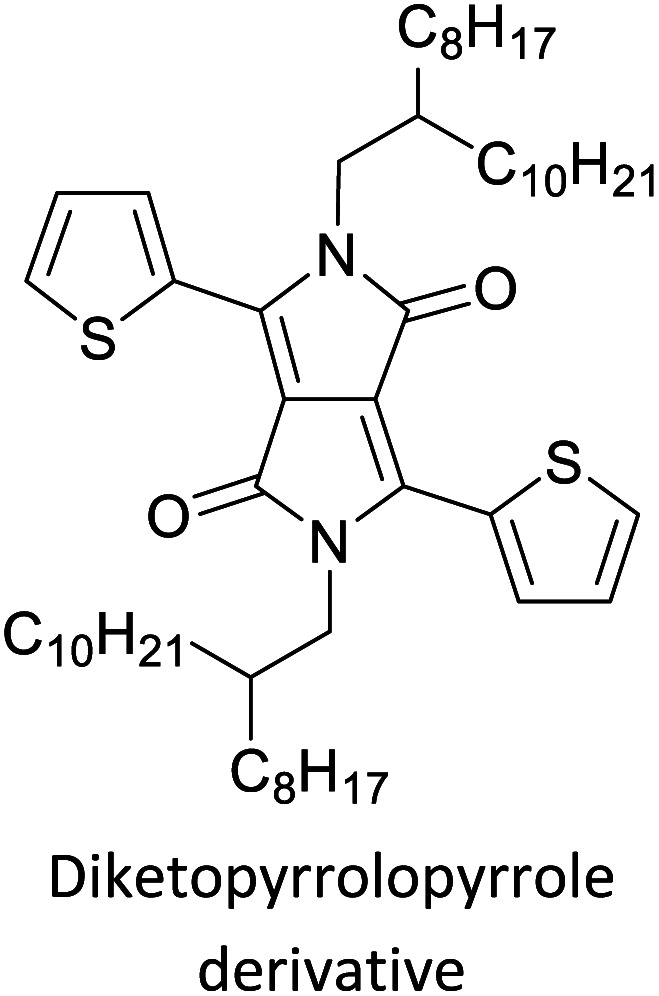	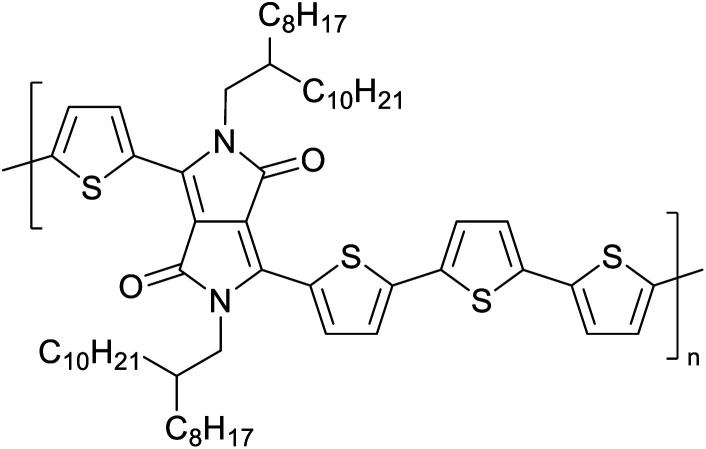	Organic field effect transistors (OFETs) and organic photovoltaics (OPVs)^66^
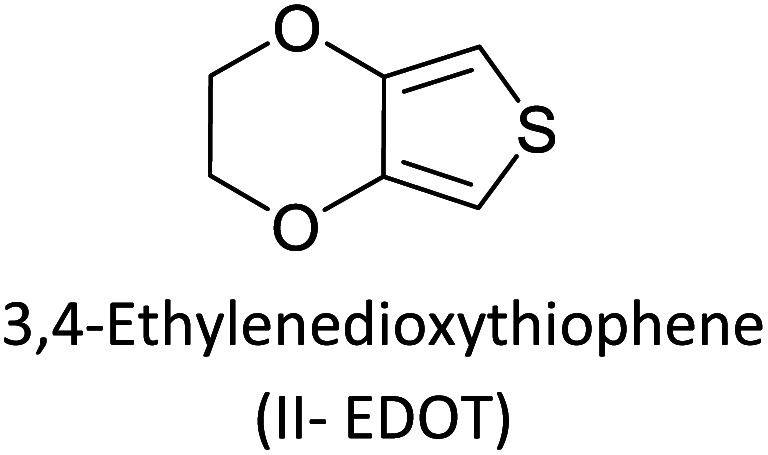	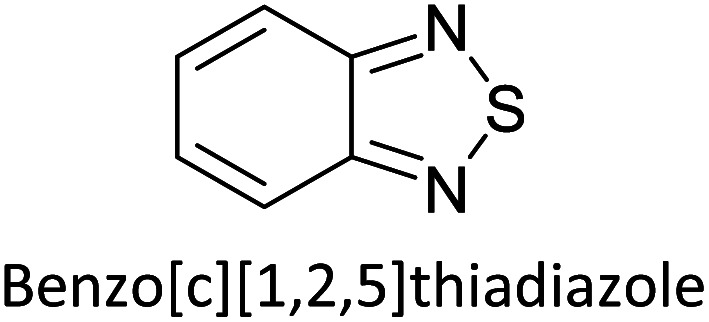	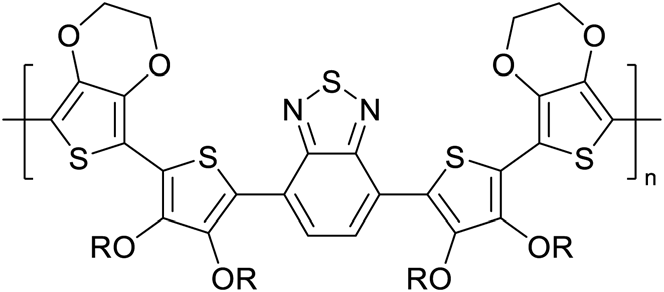	Supercapacitors^67^
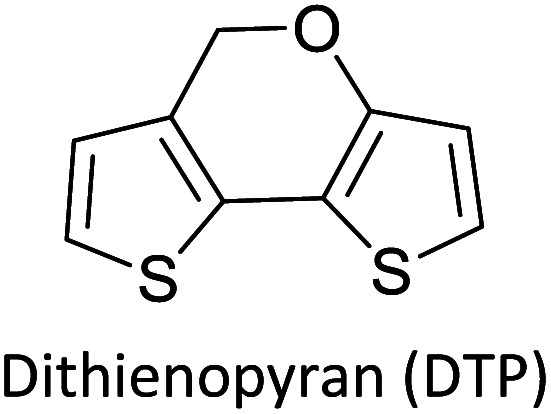	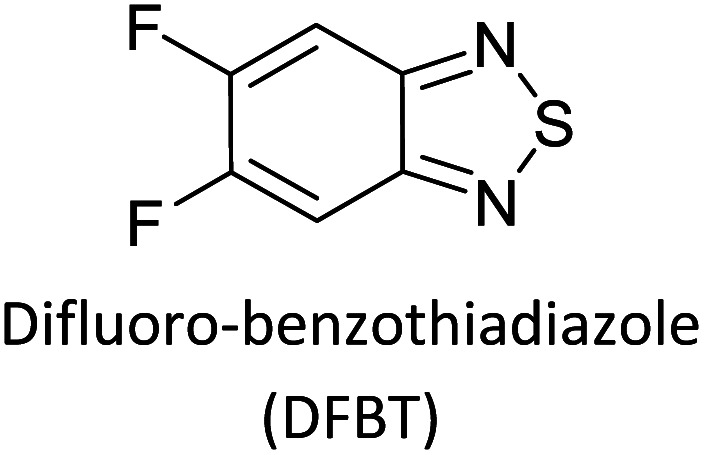	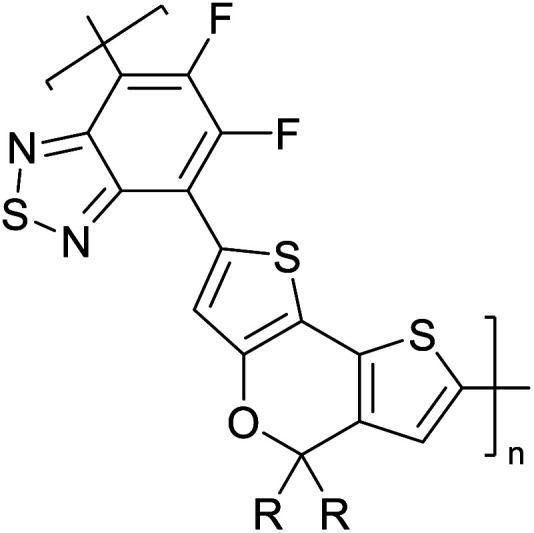	Building blocks for high performance solar cell materials^68^
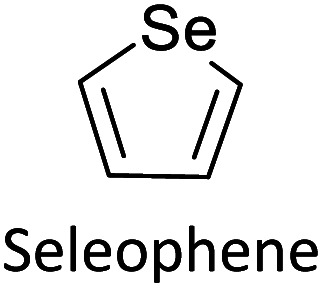	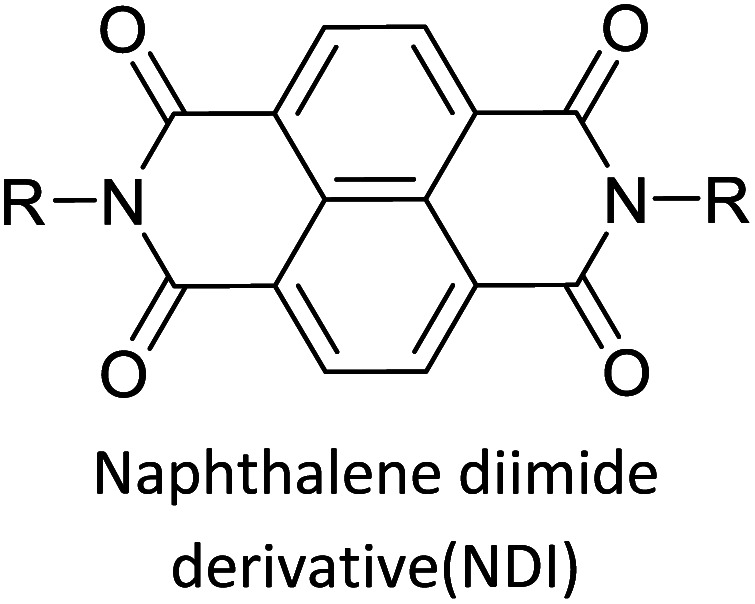	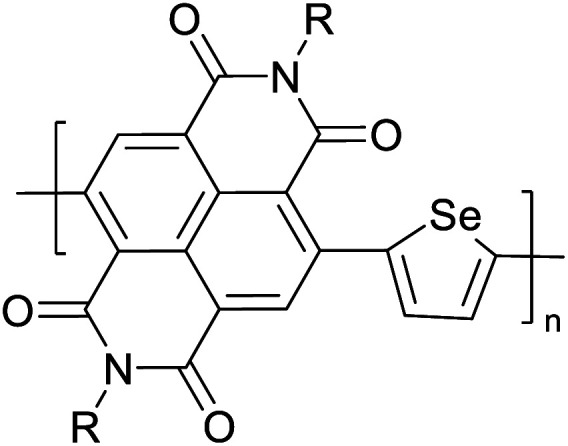	Polymer/polymer bulk heterojunction solar cells^69^
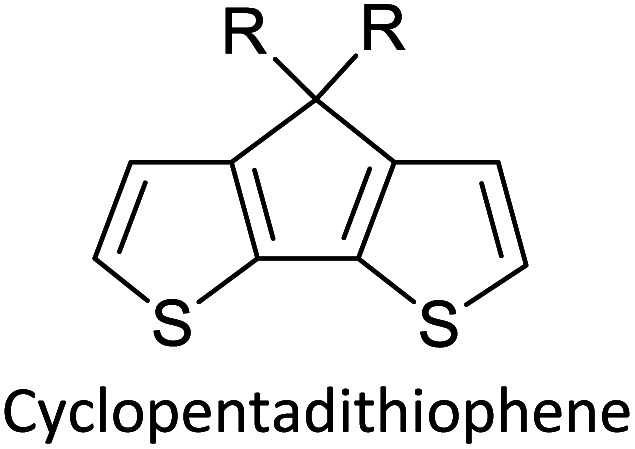	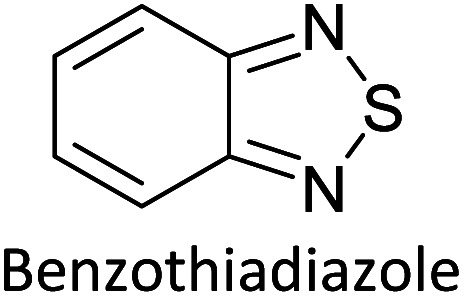	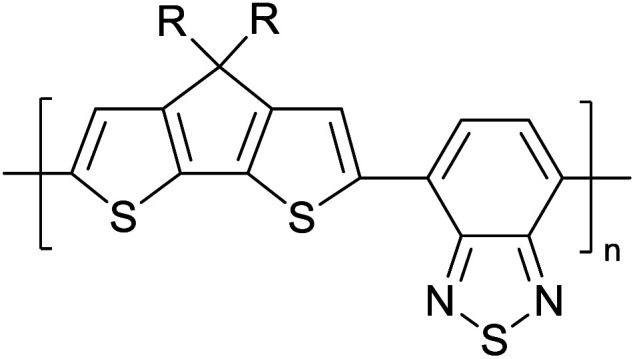	Prototypical semiconductors for high-performance field-effect transistors^70^
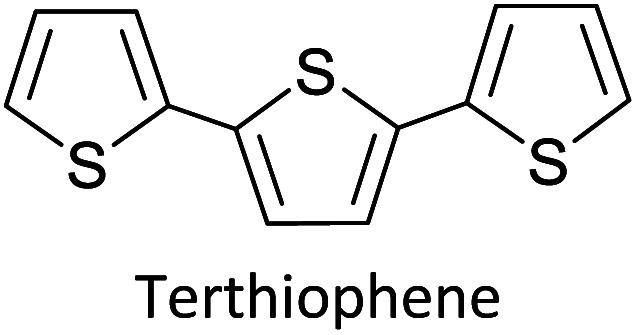	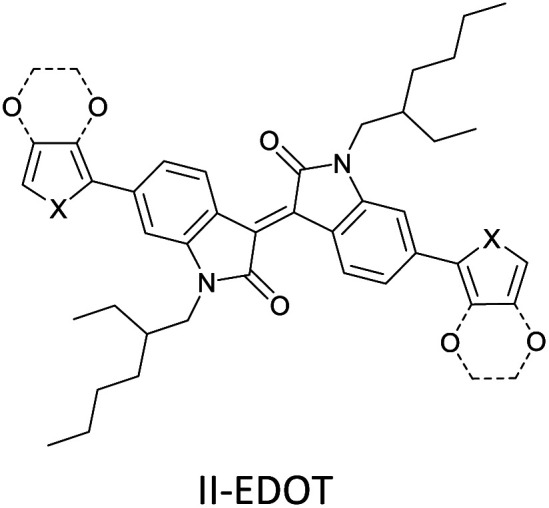	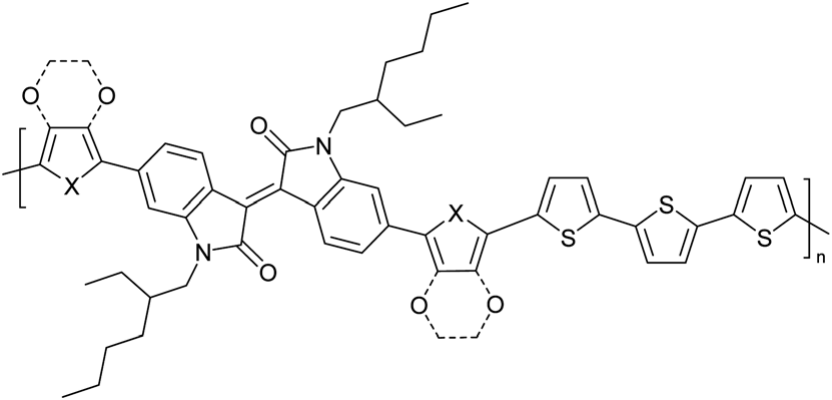	Field-effect transistors to biosensing area^71^
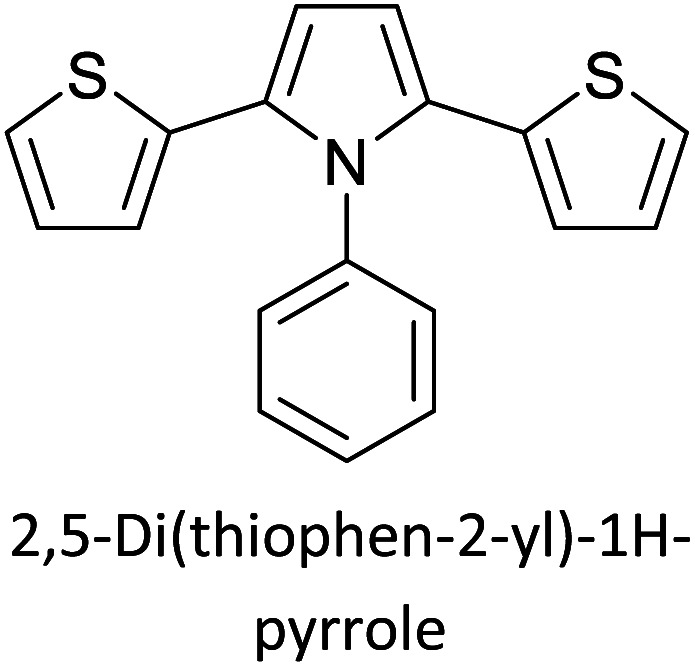	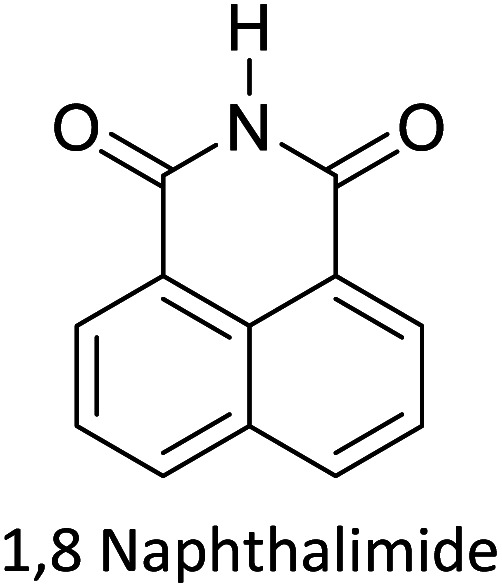	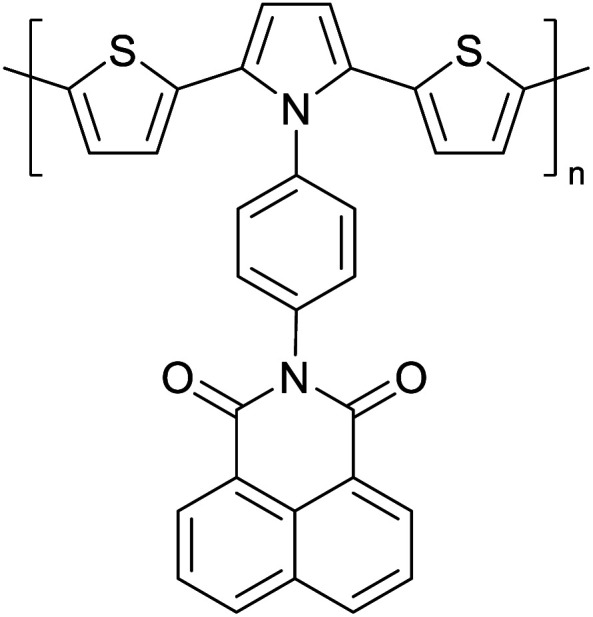	Optoelectronic applications such as OPVs, OLEDs, OFETs^72^

**Scheme 5:**
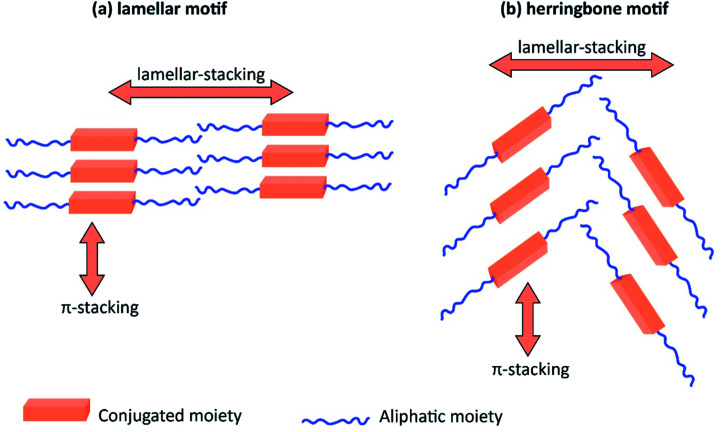
The π- and lamellar stacking for a series of naphthalene tetracarboxylic diimide (R-NDI) crystals giving rise to the lamellar motif and herringbone motif depending on the size of the aliphatic hydrocarbon moiety. This figure has been reproduced from ref. 73 with permission from *ACS Omega*, copyright 2018.

Table 3 shows the HOMO and LUMO energies and HOMO–LUMO gaps calculated through computational simulations using VASP and Gaussian 09W software by running density functional theory (DFT) with Hubbard correction for D–A type oligomers. The calculated HOMO–LUMO gaps are in the range of 1.4 eV to 2.9 eV for the examples given in Table 3. As expected, the HOMO–LUMO gap decreases with the number of D–A repeat units (*n*) is increased. Also, of interest to note is that some of the D and A molecules contain non-polar alkyl side chains such as octyl, nonyl, octyldocecyl, *etc.* and some others contain polar side chains such as ester and carboxylate functionalities. These side chains in these monomers play a crucial role by making the molecules soluble in non-polar or polar solvents. For electropolymerization, the monomers used must be soluble in a polar solvent such as acetonitrile, DMF, DMSO, *etc.* and the presence of polar side chains makes them soluble in these solvents. The non-polar side chains help the molecules soluble in non-polar solvents. Therefore, spectroscopic studies of monomers, oligomers, and even polymers in solution could be done by making them soluble. Additionally, the presence of relatively large alkyl side chains influences the solid-state packing of molecules. The π-conjugated backbone of the polymer interacts to make π–π stacking between the adjacent molecules while the non-polar side chains contribute to induced dipole-induced dipole type of interactions. The two types of interactions determine the type of packing of molecules in the solid-state. Lai *et al.* used an energy decomposition analysis called extended transition state-natural orbitals for chemical valence (ETS-NOCV) to determine π- and lamellar stacking for a series of naphthalene tetracarboxylic diimide (R-NDI) crystals.^73^ They found that the stacking principles are associated with the closest packing model where both the π-conjugated part as well as aliphatic side chains determine the packing of the molecules in the solid-state. They found that there are two stacking manners, that is, π- and lamellar stacking, in which the π-stacking occurs between conjugated groups and the lamellar stacking refers to the regular separation of the conjugated and aliphatic moieties. This has resulted in the nanoscopic phase separation of conjugated and aliphatic moieties and the formation of lamellar and herringbone motifs in the R-NDIs as shown in the Scheme 3 in ref. 23 that is reproduced as Scheme 5 here. They found that the long or bulky alkyl chains, such as C_14_H_29_, C_12_H_25_, C_6_H_13_, and 1-CH_3_C_6_H_12_, favour the formation of lamellar stacking, whereas smaller side chains such as C_5_H_11_ and C_2_H_5_ favour herringbone stacking (Scheme 5). Chen *et al.* reported the tuning of the π–π stacking distance and J-aggregation of DPP-based conjugated polymer, poly[[2,5-bis(2-octyldodecyl)-2,3,5,6-tetrahydro-3,6-dioxopyrrolo[3,4*c*] pyrrole-1,4-diyl]-*alt*-[[2,2′-(2,5-thiophene)bis-thieno[3,2-*b*]thiophen]-5,5′-diyl]] (PDPPTT-T), by introducing insulating polymer such as polystyrene (PS).^74,75^ They found that blending with PS results in the disentanglement of PDPPTT-T to cause decreased π–π stacking length and increased J-aggregation during the formation of fibrillar morphology in their fill.

**Table 3:** Computational chemical calculations of electronic properties of donor–acceptor type of electronically conducting polymers. Some alkyl sidechains attached are also given^47,48^

Donor–accepter polymer	R group	*n*	HOMO/(eV)	LUMO/(eV)	Band gap/(eV)
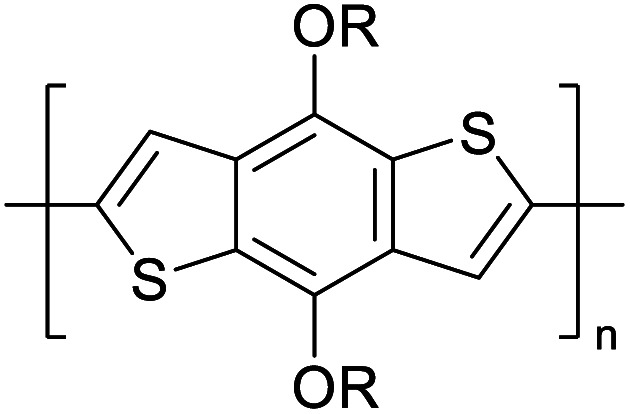	CH_3_, dodecyl	6	−5.19	−2.58	2.61
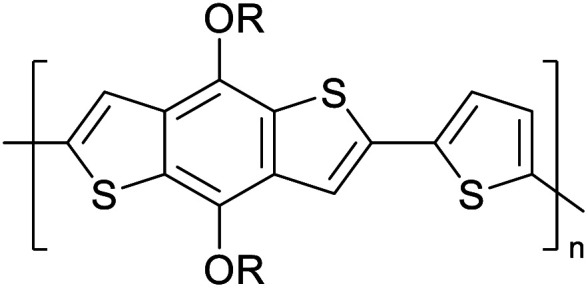	CH_3_, dodecyl	4	−5.14	−2.57	2.57
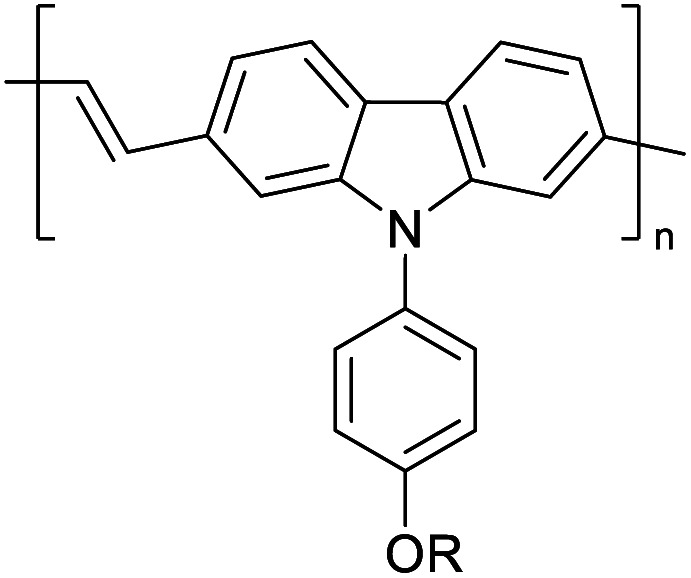	CH_3_, octyl	6	−4.81	−1.92	2.89
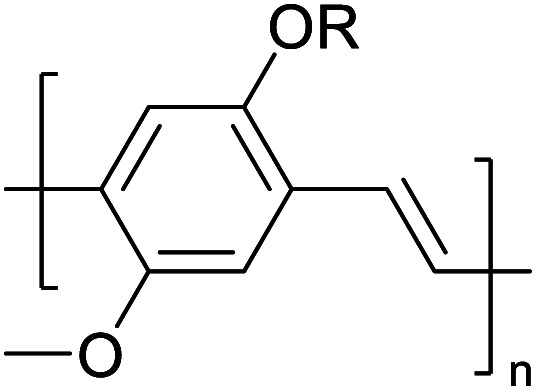	CH_3_, 2-ethylhexyl	6	−5.04	−2.39	2.65
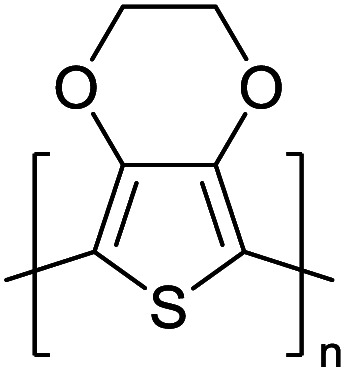		6	−4.12	−1.64	2.48
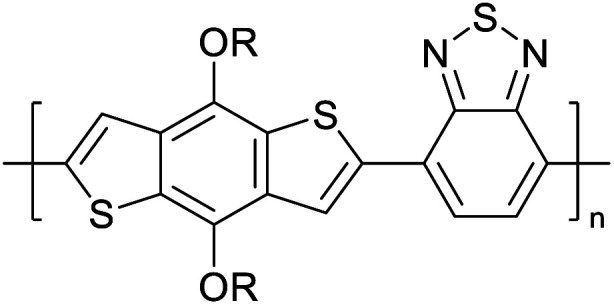	CH_3_, dodecyl	4	−5.14	−3.17	1.96
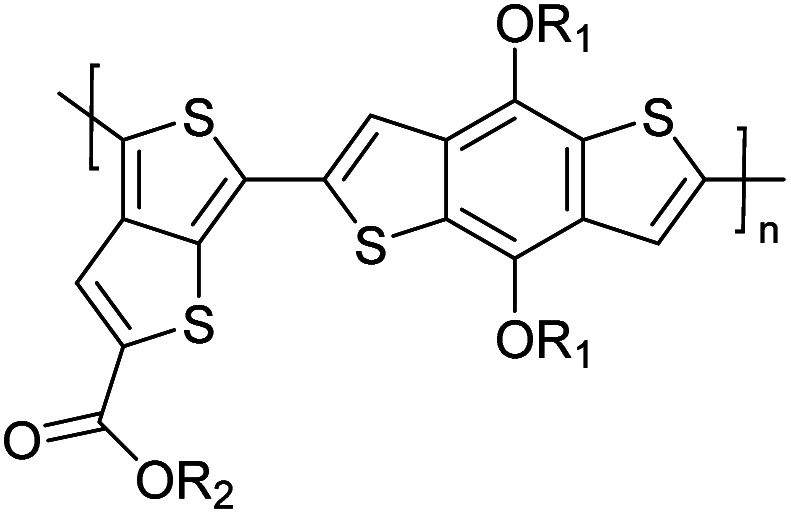	R1 = R2 = CH_3_, dodecyl	4	−5.46	−3.1	2.36
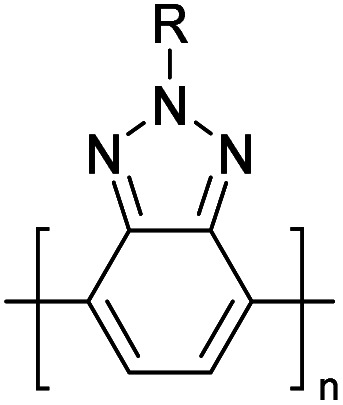	R1 = 2-ethylhexyl, R2 = octyl				
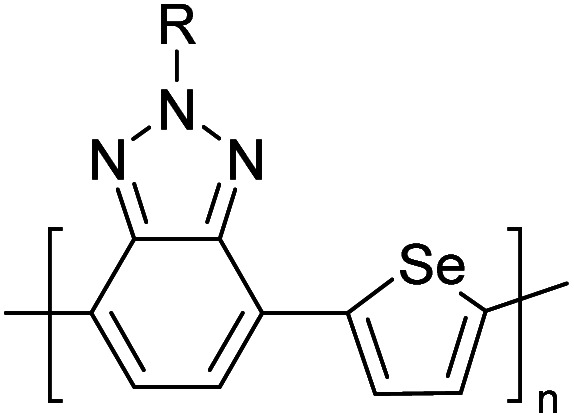	CH_3_, octyldodecyl	6	−5.03	−2.41	2.63
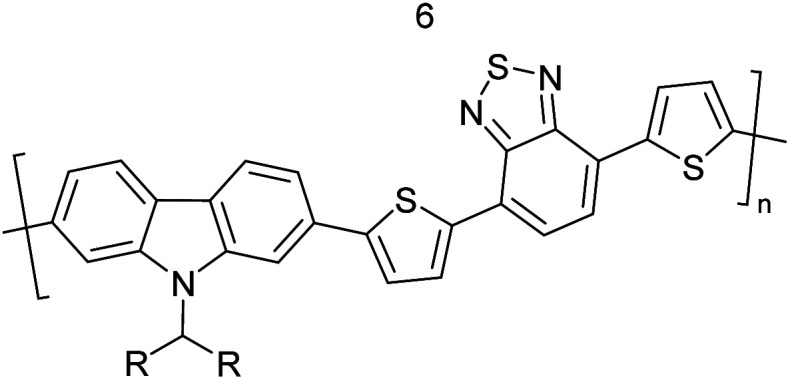	CH_3_, octyldodecyl	4	−4.88	−2.67	2.21
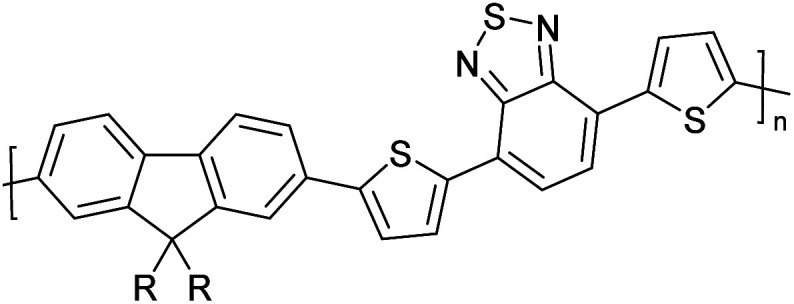	CH_3_, octyl	4	−5.04	−2.86	2.18
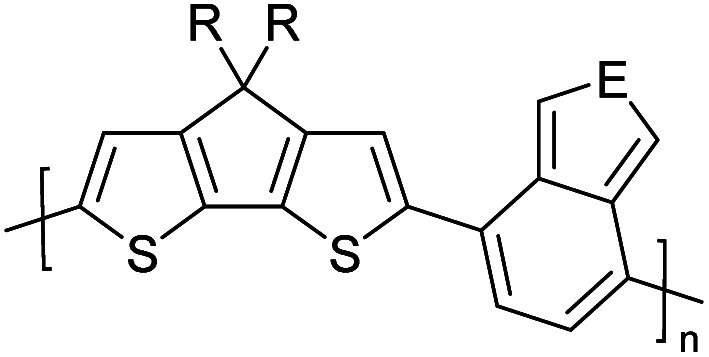	CH_3_, 3,7-dimethyloctyl	4	−5.08	−2.92	2.16
	a – R = CH_3_	4	−4.74	−3.12	1.63
	2-Ethylhexyl				
	E = S				
	b – R = CH_3_		−4.68	−3.16	1.52
	2-Ethylhexyl				
	E = Se				
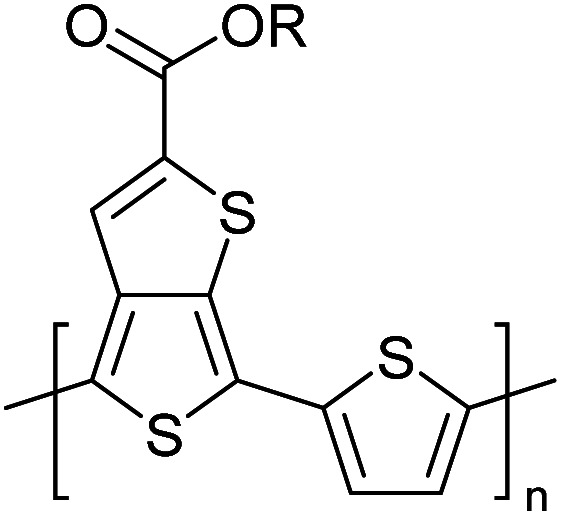	CH_3_, 1,1-dimethylnonyl	6	−4.78	−3.11	1.68
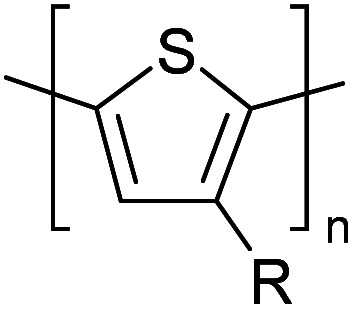	Methylthio, hexylthio	6	−5.33	−2.52	2.81
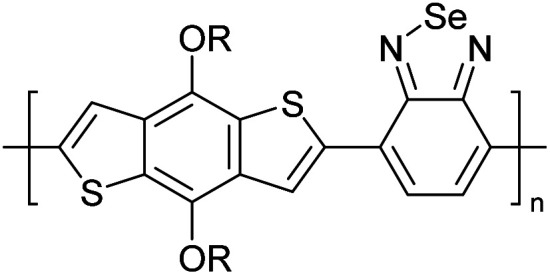	CH_3_, dodecyl	4	−5.18	−3.26	1.92
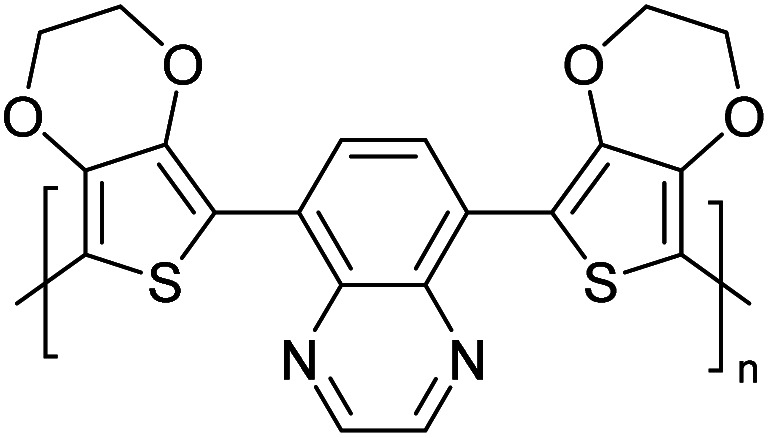	CH_3_, dodecyl	4	−5.07	−2.26	2.82
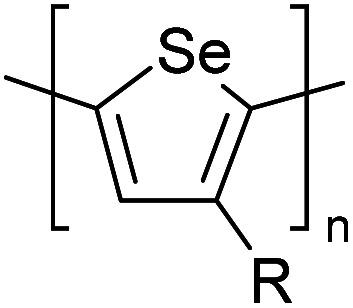	CH_3_, 1-hexyl	6	−4.77	−2.26	2.51
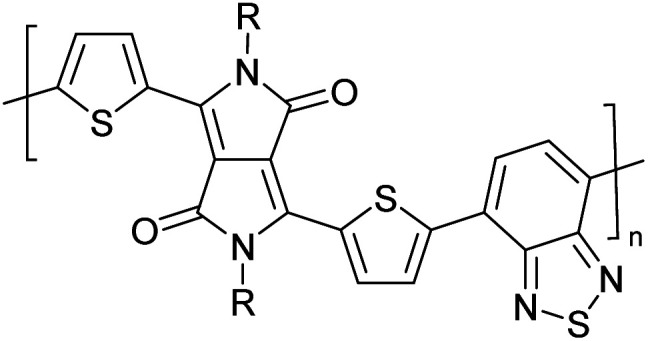	CH_3_, 2-octyldodecyl	4	−5.00	−3.45	1.55
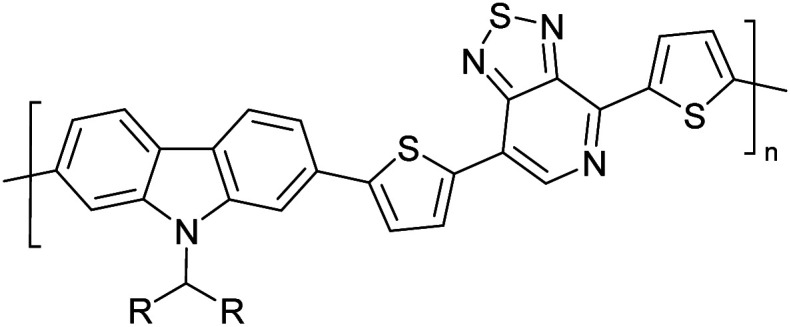	CH_3_, octyl	4	−5.14	−3.12	2.02
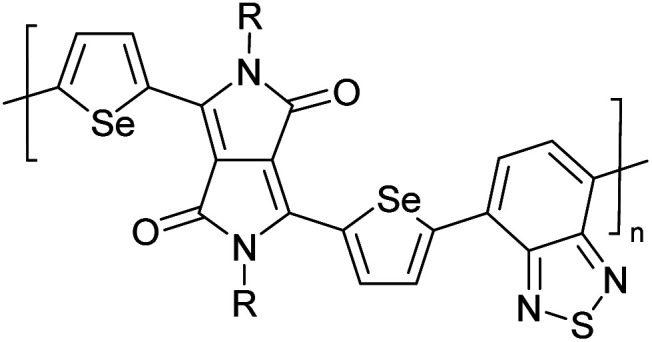	CH_3_, 2-octyldodecyl	4	−4.98	−3.52	1.47
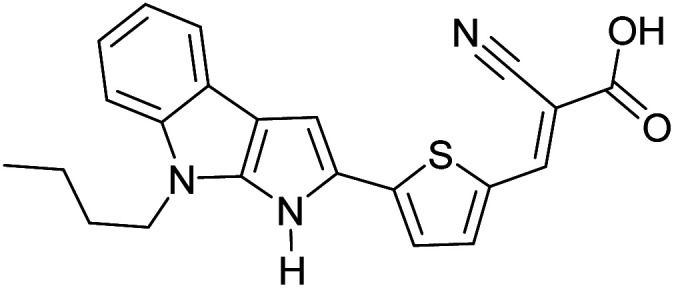		1	−5.333	−3.044	2.288
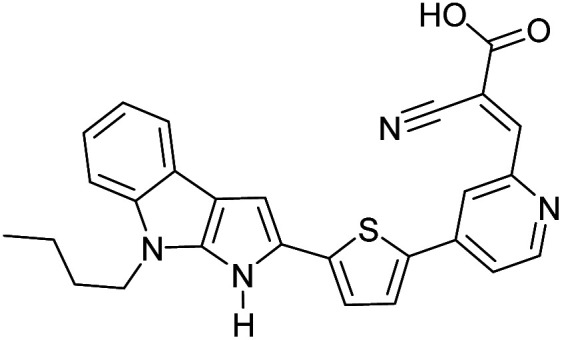		1	−5.46	−2.99	2.47

### 2.3 Electropolymerization leading to triblock and multi-block donor–acceptor type copolymers

**Fig. 6:**
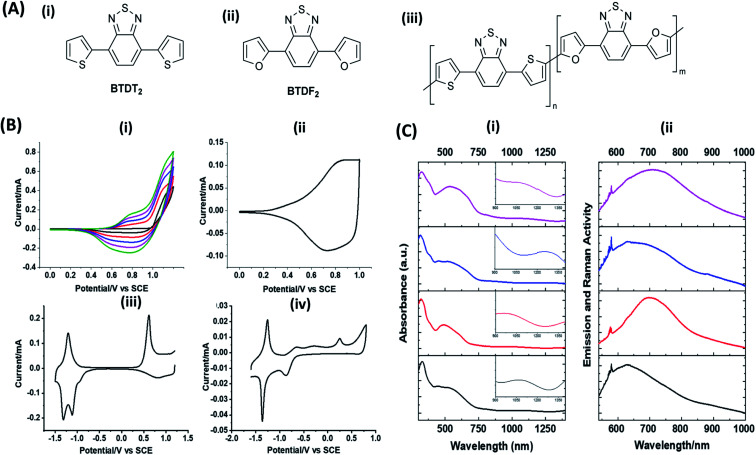
(A) The structures of (i) BTDT_2_, (ii) BTDF_2_ and (iii) the copolymers with different *m* and *n* values where *m* = *n* = 1 for the alternating copolymer, and other block copolymers prepared have the *m* : *n* values 2 : 1, 1 : 2, 3 : 2, 2 : 3 *etc.* (B) (i) Repetitive CVs for the electropolymerization of BTDT_2_ and BTDF_2_ in 1 : 1 mol ratio (2.5 mmolar each); CVs of the polymer from (ii) 0 V to +1.2 V at 100 mV s^−1^ scan rate; (iii) +1.2 V to −1.8 V for 1 : 1; and (iv) +1.2 V to −1.8 V for 2 : 5 copolymer. (C) (i) UV-visible-NIR absorption and (ii) emission and Raman spectra of BTDT_2_ (black), BTDF_2_ (red), and 1 : 1 block copolymer: p-type (blue) and n-type (purple). This figure has been reproduced from ref. 42 with permission from RSC *Journal of Material Chemistry C*, copyright 2019.

Turning to the example of copolymers of BTDT_2_ and BTDF_2_, there are T on both sides of BTD in BTDT_2_ and F on both sides of BTF_2_. The structures of the two monomers and alternating and block copolymers prepared *via* electropolymerization are given in Fig. 6(A). Fig. 6(B) (i) shows the repetitive scans for the electropolymerization of BTDT_2_ and BTDF_2_ in 1 : 1 molar ratio. The increase in current in the respective CVs and visual observation of the material deposition show that an ECP is formed on the WE surface. The CV in the BGE of the polymer from 0 to +1.0 V that is shown in b(ii) shows the typical behaviour of an ECP. Interestingly, the CVs in the range −1.8 V to +1.2 V for the polymer formed using 1 : 1 (c-iii) and 2 : 5 (c-iv) molar ratios show two distinct reduction peaks in the potential range from −1.0 V to −1.8 V which are typical of 1 − e reduction of BTD units in BTDT_2_ and BTDF_2_, respectively. The peak current ratios are 1 : 1 and 2 : 5, respectively, showing the presence of the same molar ratios taken for electropolymerization in the two cases. Additionally, the CVs of BTDT_2_ and BTDF_2_ polymers formed show only one reduction peak in this potential range and the former shows the reduction peak at a lower negative potential than that of the latter.^76,77^ All these observations clearly show the formation of copolymers with well-defined monomer ratios when a mixture of BTDT_2_ and BTDF_2_ is electropolymerized with known monomer molar ratios. Additionally, using SEM-EDAX elemental analysis, the authors have shown that the two copolymers have the above molar ratios of BTDT_2_ and BTDF_2_. In these copolymers, thiophene (T) and furan (F) act as D units and BTD as the A unit. The BTDT_2_ and BTDF_2_ polymers have two types of repeat units namely BTD and T or BTD and F. Therefore, these polymers can be considered di-block alternating copolymers. The co-polymers prepared using mixtures of BTDT_2_ and BTDF_2_ can be considered di-block copolymers of BTDT_2_ and BTDF_2_, but there are three different repeat units present since the polymers contain BTD, T, and F units. Therefore, the copolymers can be considered triblock copolymers of BTD, T and F. The ratio of the reduction peak currents of the BTD units in BTDT_2_ and BTDF_2_ is equal to the molar ratio of the two monomers used in each case thus showing the remarkable power of electropolymerization of two monomers in making stoichiometrically controlled block copolymers. Additionally, the UV-visible-NIR absorption and emission spectra reproduced in Fig. 6(C) clearly show that copolymers absorb and emit in the near and mid-infrared regions.

**Fig. 7:**
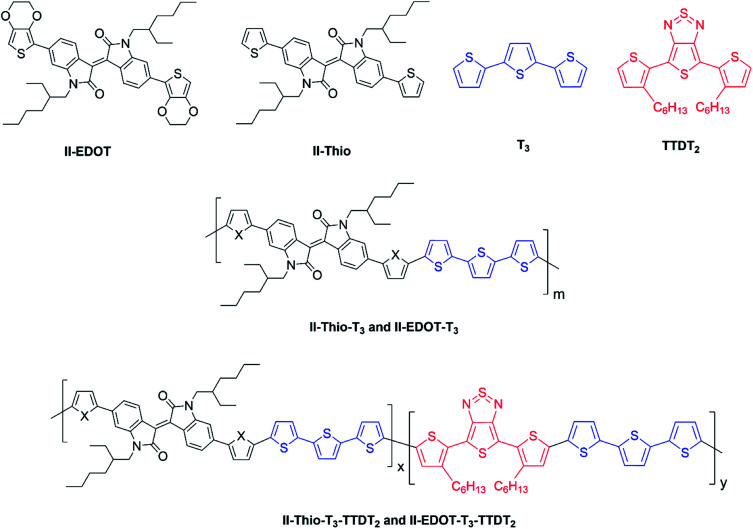
Chemical structures of the monomers used, and triblock and quadra-block copolymers prepared by electropolymerizing three monomer and four monomer systems. This figure has been reproduced from ref. 71 with permission from Chemistry Europe, copyright 2020.

The possibility for the electropolymerization of three different monomers to result in triblock copolymers was reported by Sparks *et al.* where they first published such copolymers containing several monomers in electro-copolymerization of isoindigo (iI)-based D–A polymers with intrinsically enhanced conductivity and NIR-II activity.^71^ In this work, they chemically attached EDOT or T groups on the ends of isoindigo (iI) to result in iI-EDOT_2_ and iI-T2 where the iI group possesses a solubilizing alkyl group (reproduced in Fig. 7). They chose T_3_ and TTDT_2_ as two additional monomers to electropolymerize three monomer combinations iI-EDOT_2_, TTDT_2_, T_3_ and iI-T_2_, TTDT_2_, T_3_ to result in the formation of copolymers containing all three monomer units. However, these copolymers contain several different repeat units such as iI, EDOT, T, TTD, and iI, T, TTD. Therefore, these structures can be considered as multi-block copolymers. In this study, T_3_ was shown to act as a monomer, polymerization initiator, and coupling agent to aid copolymerization. Additionally, they electropolymerized all four monomers to form multi-block copolymers with repeating units of the four different monomers. These copolymers have high electrical conductivities at all the DC potentials investigated in this research, *i.e.*, from −1.8 V to +1.0 V wrt SCE as revealed by showing the high intrinsic conductivity of the copolymers. They have also shown that as the numbers of different D and A units increase, the intrinsic conductivity increases, opening a path to electro-synthesize D–A polymers with high intrinsic electronic conductivity. Ranathunge *et al.* used benzodithiophene-*S*,*S*-tetroxide (BDTT) as electron acceptor in copolymers containing T_3_ and (EDOT)2T (EDOT-T-EDOT) *via* electropolymerization.^58^ The copolymer containing BDTT, T, and EDOT is a triblock copolymer of these materials and shows extended optical absorption towards NIR and good electronic conductivity throughout the potential range between −1.2 V to +1.0 V again indicating that D–A type copolymers have intrinsic electronic conductivity.

### 2.4 Demonstrated technological applications of donor–acceptor type copolymers

**Fig. 8:**
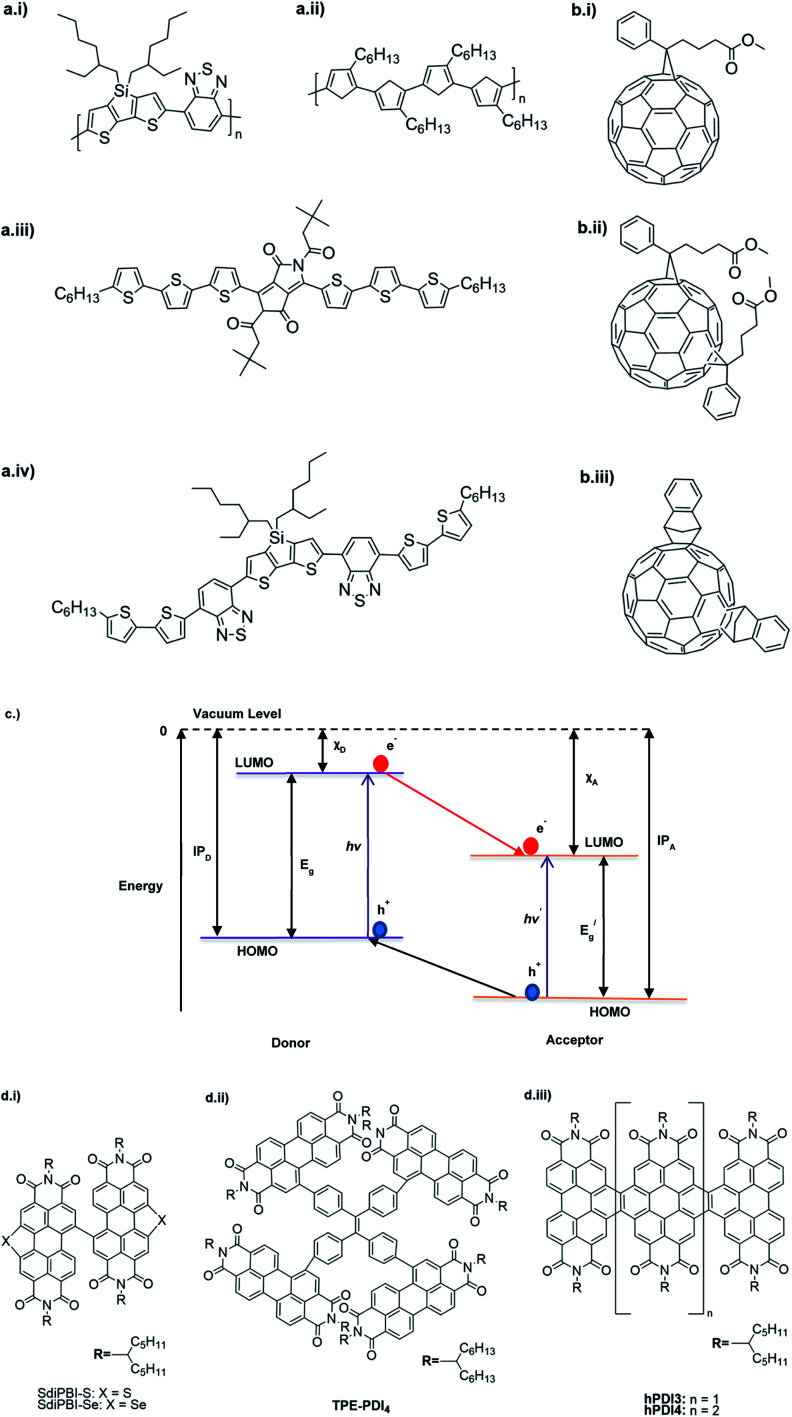
Commonly used (a) donor polymers (b) acceptor molecules both capable of absorbing radiation to generate electron–hole pairs, (c) energy level diagram showing electron–hole pair formation and separation in the donor–acceptor systems and (d) novel non-fullerene acceptor developed for use in organic solar cells. This figure has been reproduced from ref. 78 with open access permission from *Progress in Polymer Science*, copyright 2013.

The D–A type of oligomers and polymers have applications in many optoelectronic devices such as bulk-heterojunction organic solar cells (BHJOSCs) and organic field-effect transistors (OFETs). A heterojunction is defined as the contact between two materials with different electrical properties such as D–A type polymers and chemically modified fullerene derivatives. Here D–A type conjugated organic oligomers and polymers act as D moieties while fullerene derivatives act as A moieties. Chemical structures of commonly used D and A materials in BHJOSCs are shown in Fig. 8(a). When D is excited by light and injects an electron to A, it causes a negative charge in A and D becomes positively charged. The positive charges move by hole hopping, and the negative charges by electron hopping *via* charge transfer between molecules. Thus, charges move in the organic layers leading to the movement of charge in the electrodes. This is illustrated in Fig. 3 of the ref. 78 that is reproduced as Fig. 8(a).^79^ A photon with energy *hν* absorbed by D results in excitation of an electron from the HOMO to LUMO level creating a bound exciton pair of the hole (h^+^) in the HOMO and electron (e^−^) in the LUMO.^80^ The lifetime of such exciton pairs in organic semiconductors is less than 1 ns. Organic molecules usually have low dielectric constants, and hence electron–hole pair is strongly attracted by the coulombic forces making it difficult to separate under ambient conditions. As reported by Dennler *et al.* the electron–hole binding energy^80^ in organic semiconductors lie within the range of 0.35–0.50 eV and this energy is an order of magnitude greater than thermal energy under ambient conditions. Therefore, to separate h^+^–e^−^ pairs generated in D, and A should be used that should have its LUMO level below that of D and the HOMO level below that of D. Then, the e^−^ in the LUMO of D is transferred to the LUMO level of A as shown by the red arrow. However, since the HOMO level of A is below that of D the h^+^ in D cannot be transferred to the HOMO level of A and therefore it remains in the HOMO of D. This is the first step in the free carrier generation. The electron transfer should happen in a time scale less than 1 ns in order to efficiently transfer electron from the HOMO of D to that of A before the excitons recombine in D molecules. In organic semiconductors, this happens in less than 100 fs making the charge transfer very effective. Additionally, if A absorbs a photon and creates a h^+^–e^−^ pair then the energies of HOMO levels of A and D are such that h^+^ can be transferred from the HOMO level of A that of D. Therefore, holes move along the HOMO levels of D and electrons move along the LUMO levels of A when D–A type oligomers or polymers are used. Scharber and Sariciftci analysed the efficiency of BHJOSCs and plotted the increase in efficiency of the devices since 2001 to 2013 which showing a remarkable upright from 2.5% to over 10% within this period.^78^ Fullerene-bis-adducts such as those reviewed in ref. 81 were shown to give higher efficiencies. Subsequently, the use of non-fullerene acceptors (NFAs) made it possible to enhance the conversion efficiency to around 16%. Commonly used non-fullerene acceptors in BHJOSCs are perylene diimides, fused ring molecules abbreviated to Y6 and PM6 whose structures are given in Fig. 8(d) were used. Cui *et al.* reported an organic photovoltaic cell with 17% efficiency and superior processability by using A molecules with electron-withdrawing groups such as F and CN are attached to Y6 and PM6 to increase their electron deficiency and to bring their LUMO levels just below those of D molecules used in BHJOSCs. The D–A type of polymers are attractive materials for organic field effect transistors (OFETs). The OFETs are emerging as a low-cost transistor technology for creating next-generation large-area, flexible and ultra-low-cost electronics.^82–84^ Various D molecules such as benzothiadiazide (BTD), benzodithiadiazine (BBT), isoindigo (iI), their thiophene (T_0_ derivatives such as BTDT_2_, BBTT_2_, iIT_2_*etc.* together with several different A molecules including naphthaline diimide (NDI), diketopyrrolopyrrole (DPP) *etc.* have been used. The use of D–A type polymers are attractive materials for organic field effect transistors (OFETs). The OFETs are emerging as a low-cost transistor technology for creating next-generation large-area, flexible and ultra-low-cost electronics. Various D molecules such as benzothiadiazide (BTD), benzodithiadiazine (BBT), isoindigo (iI), their thiophene (T_0_ derivatives such as BTDT2, BBTT2, iIT2 *etc.* together with several different A molecules including naphthaline diimide (NDI), diketopyrrolopyrrole (DPP) *etc.* have been used. The use of D–A type molecules, oligomers and polymers enabled the improvement of charge carrier mobility from 10^−6^–10^−5^ cm V^−1^ s^−1^ to 12 cm^2^ V^−1^ s^−1^, which surpassing that of amorphous silicon (α-Si) FETs that has the charge carrier mobility in the range 0.1–1 cm^2^ V^−1^ s^−1^. Both n-type of p-type organic semiconducting polymers and ambipolar D–A polymers prepared from electrocopolymerization have been utilized. During the last thirty years, several hundreds of such organic semiconductor materials have been synthesized and used in OFETs^85^ one of the problems with high molecular weight (MW) D–A polymers is their insolubility making it difficult for solution processability. Although, low MW D–A oligomers are more soluble enabling solution processability, their charge carrier mobilities are low. Lei *et al.* developed a facile solution process which transformed a lower-MW, low-mobility diketopyrrolopyrrole-dithienylthieno[3,2-*b*]thiophene (DPP-DTT) into a high crystalline order and high-mobility semiconductor for OFETs applications. Here they used a solution fabrication of a channel semiconductor film from a lower-MW DPP-DTT and polystyrene blends to readily self-assemble and crystallize out in the polystyrene matrix as an interpenetrating, nanowire semiconductor network. This enabled to achieve significantly enhanced mobility (over 8 cm^2^ V^−1^ s^−1^), on/off ratio (107), and other desirable field-effect properties that meet impactful OFET application requirements.^86^ They are also used in electrolyte-gated organic field-effect transistors that have application in healthcare and environmental science.^87^ Recent research also show that D–A polymers such as poly(2,5-(2-decyltetradecyl)-3,6-diketopyrrolopyrrole-*alt*-5,5-(2,5-di(thien-2-yl)thieno(3,2-*b*)thiophene)) (PDPPT-TT) have applications in both field-effect transistors and in non-volatile memory devices.^88^

**Fig. 9:**
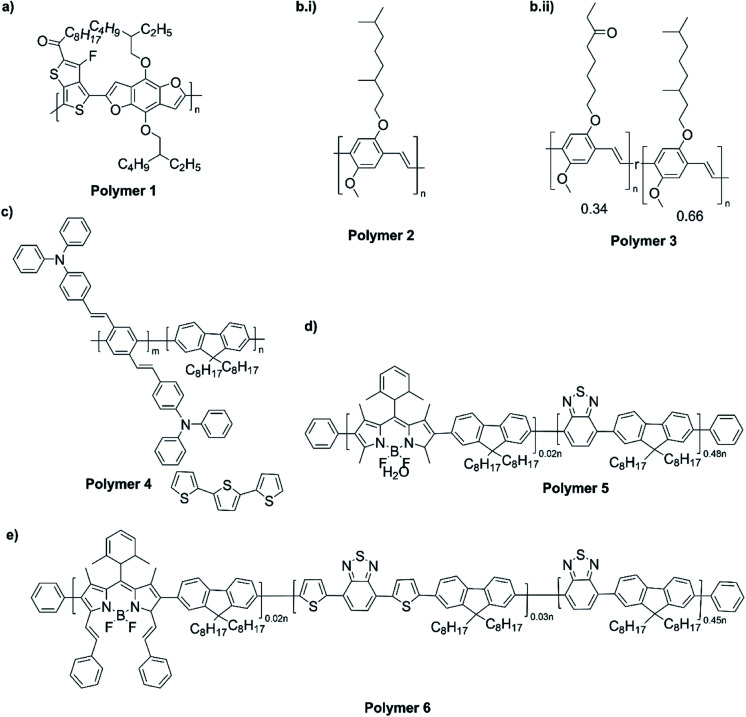
(a) A push–pull conjugated polymer based on a strong fluorinated thieno[3,4-*b*]thiophene acceptor and benzadifuran based donor (polymer 1). (b) A polymer with chromophores comprising of fluorene-based CP with a perylene diimide (PDI) dye (polymer 2). (c) PPV-based NPs which emit at 580 nm when excited by 830 nm that are used two-photon excitation (polymers 3 and 4). (d) Boron dipyrromethene (BODIPY) based D–A polymers that have low emission bandwidths (emission band widths at half maximum of only 40–55 nm) that are used for simultaneous detection of multiple targets (polymers 5 and 6). (e) Finalized polymer after combining polymer 4 and polymer 5. This figure has been reproduced from ref. 91 with permission from *ACS Applied Materials and Interfaces*, copyright 2015.

The NIR absorption and emission of D–A type of ECPs have been utilized in nanoprobes for activatable NIR-II fluorescence imaging.^89^ These polymers are used in bio-imaging due to several reasons such as their high fluorescence brightness, high photostability, fast emission rates, non-blinking behaviour and low cytotoxicity. A review by Braeken *et al.* describes how these materials can be made to possess hydrophilicity for increased bioavailability by making their nanoparticles.^90^ The D–A polymers based on fluorene, copolymerized together with benzothiadiazole, quinoxaline and/or thiophene have shown strong absorption and emission in the NIR region. A push–pull conjugated polymer based on a strong fluorinated thieno[3,4-*b*]thiophene acceptor and benzadifuran based donor shown in Fig. 9 (polymer 1) has an absorption in the near-infrared region because of the strong donor–strong acceptor approach. They prepared polymer nanoparticles (NPs) stabilized by the PEGylated (PEG = polyethylene glycol) surfactant *via* the mini-emulsion technique. These polymer NPs have absorption at *λ* = 654 nm, and emission in the NIR region peaking at *λ* = 1047 nm. The emission in NIR is particularly useful for bioimaging because of a lower autofluorescence and reduced photon scattering in biological tissues, resulting in a higher spatial resolution and deeper tissue penetration. One of the drawbacks of these nanoparticles is their poor photoluminescence quantum yield PLQY where it is 1.7% for this copolymer. To overcome this, D–A polymers have been developed to use in two-photon excitation microscopy^91^ where chromophores comprising of fluorene-based CP with a perylene diimide (PDI) dye Fig. 9 (polymer 2) creating particles that can be excited at *λ* = 800 nm, while emission occurs at *λ* = 730 nm. Peters *et al.* used two-photon excitation of PPV-based NPs which emit at 580 nm when excited by 830 nm^92^Fig. 9 (polymers 3 and 4). The use of these materials enables the excitation in the NIR region for emission to occur in the visible region to show the characteristic colours of the emission. Polymers 5 and 6 shown in Fig. 9 are those developed by Rong *et al.*^93^ and are boron dipyrromethene (BODIPY) based D–A polymers that have low emission band widths (emission band widths at half maximum of only 40–55 nm). They are suitable for use in the simultaneous detection of multiple targets. One of the problems with D–A type conjugated polymers is their π–π stacking leading to fluorescence quenching. Several approaches are used to overcome this problem: the use of bulky alkyl sidechains to maintain a sufficiently large distance between molecules and to introduce steric hindrance to avoid close contact leading to stacking,^94–97^ the introduction of small functional side chains,^98^ co-precipitation with other polymers such as PEG and freezing of CPNPs to their relaxed states.^99,100^ The utilization of these technologies enabled significant enhancement in FQYs to around 46% thus making these materials ideal for bio-imaging^101^ applications.

## 3. Summary

This review on electropolymerization is a useful method for the synthesis of complicated ECPs with intrinsic electronic conductivity due to the presence of energy matching donor and acceptor moieties. The review identified the basics of electropolymerization leading to second-generation electronically conducting polymers followed by its application in complex push–pull type donor–acceptor ECPs. The literature pertinent to the synthesis of D–A type ECPs from 2004 to 2021 has been tabulated and some illustrative examples described. Finally, applications of D–A type polymers in optoelectronics, such as organic solar cells, organic field-effect transistors and bioimaging were presented.

## 4. Conclusions

Recent advances in electropolymerization leading to stoichiometrically well-defined block copolymers containing conjugated donor and acceptor moieties to result in intrinsic electronic conductivity and extended optical absorption and emission towards the near infrared to mid infrared regions have been critically reviewed condensing the literature available from 2004 tom 2021. Demonstrated technological applications of the materials were also discussed. To understand the complex chemistry of donor–acceptor type of polymers, fundamental aspects of electropolymerization leading to common second generation electronically conducting polymers were presented at the beginning. This critical review will be useful for researchers, graduate students and others who are working on electronically conducting polymer research and development studies.

In this work, the electropolymerization is shown to be a convenient and fast technique for the preparation of complex organic electronically conducting polymers with monomers containing donor and acceptor moieties. Further, this technique can be used to synthesize block copolymers of this type with different monomers. Our current research is focused on isolating conjugated monomers containing donor and acceptor molecules within the same molecule such as berberine from natural products and to use them in making complex donor–acceptor type of electronically conducting polymers. In this way, tedious synthetic approaches to make complex monomers can be avoided and the Nature's unique selections can be harnessed to make advanced materials with inherent properties suitable for opto-electronic devices and in biomarking.

## Author contributions

The senior academics (Professors R. M. G. Rajapakse, Davita L. Watkins, Daniel R. Strongin, Nuwan Harsha Attanayake, Benjamin R. Horrocks and Dhayalan Velauthapillai) were involved in assimilation and organization of the structure and the content of the manuscript. Research students (Tharindu A. Ranathunge, A. U. Malikaramage, H. M. N. P. Gunarathna, Lahiru Sandakelum, Shane Wylie, P. G. P. R. Abewardana, M. G. S. A. M. E. W. D. D. K. Egodawele, W. H. M. R. N. K. Herath, Sanjaya V. Bandara) were involved in organizing the data from literature, arranging figures and tables, and understanding concepts. As such, all the authors have contributed significantly to this manuscript.

## Conflicts of interest

There are no conflicts to declare.

## Supplementary Material

## References

[cit1] Kanazawa K. K., Diaz A. F., Geiss R. H., Gill W. D., Kwak J. F., Logan J. A., Rabolt J. F., Street G. B. (1979). J. Chem. Soc., Chem. Commun..

[cit2] Bartlett P. N., Birkin P. R. (1993). Synth. Met..

[cit3] Chiang J. C., MacDiarmid A. G. (1986). Synth. Met..

[cit4] MacDiarmid A. G. (2010). Chem. Inf..

[cit5] Negi Y. S., Adhyapak P. V. (2007). J. Macromol. Sci., Part C: Polym. Rev..

[cit6] Tourillon G., Garnier F. (1982). J. Electroanal. Chem. Interfacial Electrochem..

[cit7] Katz H. E., Searson P. C., Poehler T. O. (2010). J. Mater. Res..

[cit8] Ghosh S., Inganäs O. (2000). Electrochem. Solid-State Lett..

[cit9] Gogoi R. , Dutt S. and Siril P. F., Conjugated Polymer Nanostructures for Energy Conversion and Storage Applications, 2021, pp. 267–296

[cit10] Shinkle A. A., Pomaville T. J., Sleightholme A. E. S., Thompson L. T., Monroe C. W. (2014). J. Power Sources.

[cit11] Tsierkezos N. G., Philippopoulos A. I. (2009). Fluid Phase Equilib..

[cit12] Ue M., Takeda M., Takehara M., Mori S. (1997). J. Electrochem. Soc..

[cit13] Data P., Pander P., Lapkowski M., Swist A., Soloducho J., Reghu R. R., Grazulevicius J. v. (2014). Electrochim. Acta.

[cit14] Xiao Y. M., Lin J. Y., Wu J. H., Tai S. Y., Yue G. T. (2012). Electrochim. Acta.

[cit15] Sanchis-Gual R., Seijas-Da Silva A., Coronado-Puchau M., Otero T. F., Abellán G., Coronado E. (2021). Electrochim. Acta.

[cit16] Sarkar S., Ramarao S. D., Das T., Das R., Vinod C. P., Chakraborty S., Peter S. C. (2021). ACS Catal..

[cit17] Deng P., Liu L., Ren S., Li H., Zhang Q. (2012). Chem. Commun..

[cit18] Bouzzine S. M., Salgado-Morán G., Hamidi M., Bouachrine M., Pacheco A. G., Glossman-Mitnik D. (2015). J. Chem..

[cit19] Polozhentseva J., Novozhilova M., Karushev M. (2022). Int. J. Mol. Sci..

[cit20] Budkov Y. A., Kalikin N. N., Kolesnikov A. L. (2022). Phys. Chem. Chem. Phys..

[cit21] Ma L., Li Z., Chen B., Xue P., Wang Z., Wu Y., Zhan X., Liu Y., Chen X. (2022). Adv. Electron. Mater..

[cit22] Seo K.-D., Park D.-S., Shim Y.-B. (2022). J. Electrochem. Soc..

[cit23] Camarada M. B., Jaque P., Díaz F. R., del Valle M. A. (2011). J. Polym. Sci., Part B: Polym.
Phys..

[cit24] Ocheje M. U., Comí M., Yang R., Chen Z., Liu Y., Yousefi N., Al-Hashimi M., Rondeau-Gagné S. (2022). J. Mater. Chem. C.

[cit25] Soroceanu M., Constantin C.-P., Damaceanu M.-D. (2022). Prog. Org. Coat..

[cit26] Karpagam S., Anupriya P., Supraja N. (2021). Polym. Bull..

[cit27] Hall N. (2003). Chem. Commun..

[cit28] Heeger A. J. (2001). Rev. Mod. Phys..

[cit29] Shirakawa H. (2001). Rev. Mod. Phys..

[cit30] MacDiarmid A. G. (2001). Rev. Mod. Phys..

[cit31] Swager T. M. (2017). Macromolecules.

[cit32] Kane-Maguire L. A. P., Wallace G. G. (2010). Chem. Soc. Rev..

[cit33] Ibanez J. G., Rincón M. E., Gutierrez-Granados S., Chahma M., Jaramillo-Quintero O. A., Frontana-Uribe B. A. (2018). Chem. Rev..

[cit34] Yang Y., da Costa R. C., Smilgies D. M., Campbell A. J., Fuchter M. J. (2013). Adv. Mater..

[cit35] Zhang D., Zhang L., Wang B., Piao G. (2013). J. Mater..

[cit36] de Lacy Costello B. P. J., Ratcliffe N. M., Sivanand P. S. (2003). Synth. Met..

[cit37] Baker C., Wagner K., Wagner P., Officer D. L., Mawad D. (2021). Adv. Phys.: X.

[cit38] Zhou C., Sun X., Han J. (2020). Mater. Chem. Front..

[cit39] Pavel I.-A., Lakard S., Lakard B. (2022). Chemosensors.

[cit40] Tang Y., Jin S., Zhang S., Wu G.-Z., Wang J.-Y., Xu T., Wang Y., Unruh D., Surowiec K., Ma Y., Wang S., Katz C., Liang H., Li Y., Cong W., Li G. (2022). Research.

[cit41] Mouffouk F., Brown S. J., Demetriou A. M., Higgins S. J., Nichols R. J., Rajapakse R. M. G., Reeman S. (2005). J. Mater. Chem..

[cit42] Rajapakse R. M. G., Attanayake N. H., Karunathilaka D., Steen A. E., Hammer N. I., Strongin D. R., Watkins D. L. (2019). J. Mater. Chem. C.

[cit43] Hass R., García-Cañadas J., Garcia-Belmonte G. (2005). J. Electroanal. Chem..

[cit44] Zayat B., Das P., Thompson B. C., Narayan S. R. (2021). J. Phys. Chem. C.

[cit45] Velauthamurty K., Higgins S. J., Rajapakse R. M. G., Bandara H. M. N., Shimomura M. (2010). Electrochim. Acta.

[cit46] Luo X., Shen H., Perera K., Tran D. T., Boudouris B. W., Mei J. (2021). ACS Macro Lett..

[cit47] Åkerlund L., Emanuelsson R., Hernández G., Ruipérez F., Casado N., Brandell D., Strømme M., Mecerreyes D., Sjödin M. (2019). ACS Appl. Energy Mater..

[cit48] Velauthamurty K., Rajapakse R. M. G., Higgins S. J. (2017). Inorg. Chim. Acta.

[cit49] Suriyakumar S., Bhardwaj P., Grace A. N., Stephan A. M. (2021). Batteries Supercaps.

[cit50] Hayashi S., Koizumi T. (2012). Polym. Chem..

[cit51] Liaw D. J., Wang K. L., Kang E. T., Pujari S. P., Chen M. H., Huang Y. C., Tao B. C., Lee K. R., Lai J. Y. (2009). J. Polym. Sci., Part A: Polym. Chem..

[cit52] Helten H. (2016). Chem.–Eur. J..

[cit53] Kularatne R. S., Taenzler F. J., Magurudeniya H. D., Du J., Murphy J. W., Sheina E. E., Gnade B. E., Biewer M. C., Stefan M. C. (2013). J. Mater. Chem. A.

[cit54] Salzner U., Karalti O., Durdaǧi S. (2006). J. Mol. Model..

[cit55] Zhu Z., Waller D., Gaudiana R., Morana M., Mühlbacher D., Scharber M., Brabec C. (2007). Macromolecules.

[cit56] Beaujuge P. M., Ellinger S., Reynolds J. R. (2008). Nat. Mater..

[cit57] Nakashima M., Otsura T., Naito H., Ohshita J. (2015). Polym. J..

[cit58] Ranathunge T. A., Nirmani L. P. T., Nelson T. L., Watkins D. L. (2021). ChemElectroChem.

[cit59] Nielsen C. B., Turbiez M., McCulloch I. (2013). Adv. Mater..

[cit60] Zhang D., Wang M., Liu X., Zhao J. (2016). RSC Adv..

[cit61] Cheng X., Ma Y., Ju X., Zhao W., Zhao J., Li Q., Sang Z., Du H., Zhang Y. (2020). Synth. Met..

[cit62] Song H., Deng Y., Gao Y., Jiang Y., Tian H., Yan D., Geng Y., Wang F. (2017). Macromolecules.

[cit63] Feng F., Kong L., Du H., Zhao J., Zhang J. (2018). Polymers.

[cit64] Nakamura T., Ishikura Y., Arakawa N., Hori M., Satou M., Endo M., Masui H., Fuse S., Takahashi T., Murata Y., Murdey R., Wakamiya A. (2019). RSC Adv..

[cit65] Ranathunge T. A., Ngo D. T., Karunarathilaka D., Attanayake N. H., Chandrasiri I., Brogdon P., Delcamp J. H., Rajapakse R. M. G., Watkins D. L. (2020). J. Mater. Chem. C.

[cit66] Liu F., Wang C., Baral J. K., Zhang L., Watkins J. J., Briseno A. L., Russell T. P. (2013). J. Am. Chem. Soc..

[cit67] Ming S., Li Z., Zhen S., Liu P., Jiang F., Nie G., Xu J. (2020). Chem. Eng. J..

[cit68] Dou L., Chen C. C., Yoshimura K., Ohya K., Chang W. H., Gao J., Liu Y., Richard E., Yang Y. (2013). Macromolecules.

[cit69] Earmme T., Hwang Y. J., Murari N. M., Subramaniyan S., Jenekhe S. A. (2013). J. Am. Chem. Soc..

[cit70] Li M., An C., Pisula W., Müllen K. (2018). Acc. Chem. Res..

[cit71] Sparks N. E., Ranathunge T. A., Attanayake N. H., Brodgon P., Delcamp J. H., Rajapakse R. M. G., Watkins D. L. (2020). ChemElectroChem.

[cit72] Oguzhan E., Bilgili H., Baycan Koyuncu F., Ozdemir E., Koyuncu S. (2013). Polymer.

[cit73] Lai Y. Y., Huang V. H., Lee H. T., Yang H. R. (2018). ACS Omega.

[cit74] Chen L., Wang H., Liu J., Xing R., Yu X., Han Y. (2016). J. Polym. Sci., Part B: Polym. Phys..

[cit75] McCormick T. M., Bridges C. R., Carrera E. I., Dicarmine P. M., Gibson G. L., Hollinger J., Kozycz L. M., Seferos D. S. (2013). Macromolecules.

[cit76] Rajapakse R. M. G., Attanayake N. H., Karunathilaka D., Steen A. E., Hammer N. I., Strongin D. R., Watkins D. L. (2019). J. Mater. Chem. C.

[cit77] Jarosz T., Stolarczyk A., Glosz K. (2020). Curr. Org. Chem..

[cit78] Scharber M. C., Sariciftci N. S. (2013). Prog. Polym. Sci..

[cit79] Kularatne R. S., Magurudeniya H. D., Sista P., Biewer M. C., Stefan M. C. (2013). J. Polym. Sci., Part A: Polym. Chem..

[cit80] Dennler G., Scharber M. C., Brabec C. J. (2009). Adv. Mater..

[cit81] Li Y. (2013). Chem.–Asian J..

[cit82] Yeo J., Hong S., Lee D., Hotz N., Lee M. T., Grigoropoulos C. P., Ko S. H. (2012). PLoS One.

[cit83] Di C. A., Zhang F., Zhu D. (2013). Adv. Mater..

[cit84] Mei Y. (2019). Nanoelectronics.

[cit85] Xia X., Lei T., Pei J., Liu C. (2014). Chin. J. Org. Chem..

[cit86] Lei Y., Deng P., Li J., Lin M., Zhu F., Ng T. W., Lee C. S., Ong B. S. (2016). Sci. Rep..

[cit87] Doumbia A., Tong J., Wilson R. J., Turner M. L. (2021). Adv. Electron. Mater..

[cit88] Choi Y. J., Kim J., Kim M. J., Ryu H. S., Woo H. Y., Cho J. H., Kang J. (2021). Micromachines.

[cit89] Tang Y., Li Y., Lu X., Hu X., Zhao H., Hu W., Lu F., Fan Q., Huang W. (2019). Adv. Funct. Mater..

[cit90] Braeken Y., Cheruku S., Ethirajan A., Maes W. (2017). Materials.

[cit91] Lv Y., Liu P., Ding H., Wu Y., Yan Y., Liu H., Wang X., Huang F., Zhao Y., Tian Z. (2015). ACS Appl. Mater. Interfaces.

[cit92] Peters M., Zaquen N., D'Olieslaeger L., Bové H., Vanderzande D., Hellings N., Junkers T., Ethirajan A. (2016). Biomacromolecules.

[cit93] Rong Y., Wu C., Yu J., Zhang X., Ye F., Zeigler M., Gallina M. E., Wu I. C., Zhang Y., Chan Y. H., Sun W., Uvdal K., Chiu D. T. (2013). ACS Nano.

[cit94] Liu H. Y., Wu P. J., Kuo S. Y., Chen C. P., Chang E. H., Wu C. Y., Chan Y. H. (2015). J. Am. Chem. Soc..

[cit95] Chen C. P., Huang Y. C., Liou S. Y., Wu P. J., Kuo S. Y., Chan Y. H. (2014). ACS Appl. Mater. Interfaces.

[cit96] Liu J., Feng G., Ding D., Liu B. (2013). Polym. Chem..

[cit97] Liu J., Li K., Liu B. (2015). Adv. Sci..

[cit98] D'Olieslaeger L., Braeken Y., Cheruku S., Smits J., Ameloot M., Vanderzande D., Maes W., Ethirajan A. (2017). J. Colloid Interface Sci..

[cit99] il Kim B. S., Jin Y. J., Lee W. E., Byun D. J., Yu R., Park S. J., Kim H., Song K. H., Jang S. Y., Kwak G. (2015). Adv. Opt. Mater..

[cit100] Ding D., Liu J., Feng G., Li K., Hu Y., Liu B. (2013). Small.

[cit101] Ni Y., Wu J. (2014). Org. Biomol. Chem..

